# A small molecule directly targets NLRP3 to promote inflammasome activation and antitumor immunity

**DOI:** 10.1038/s41419-025-07578-0

**Published:** 2025-04-04

**Authors:** Xuemei Liu, Hongbin He, Minghui Qi, Zhongjun Jiang, Bolong Lin, Xiaqiong Wang, Di Wang, Ming Ma, Wei Jiang, Rongbin Zhou

**Affiliations:** 1 National Key Laboratory of immune response and immunotherapy, Center for Advanced Interdisciplinary Science and Biomedicine of IHM, School of Basic Medical Sciences, Division of Life Sciences and Medicine, University of Science and Technology of China, Hefei, Anhui China; 2 Institute of Immunology, Zhejiang University School of Medicine, Hangzhou, China

**Keywords:** Immunotherapy, Inflammasome, Tumour immunology

## Abstract

Immune checkpoint blockade (ICB) therapies have emerged as promising treatment of cancer, but the efficacy is limited. NLRP3 inflammasome activation in tumor microenvironment can promote the infiltration of cytotoxic lymphocytes and antitumor immunity, but it is unclear whether ICB resistance can be overcome by directly targeting NLRP3. Here we show that a small molecule compound directly targeting NLRP3 can induce inflammasome activation and anti-tumor immunity. 2-guanidinobezimidazole (2GBI) directly bound to NLRP3 and induced inflammasome activation, which was independent of potassium efflux, chloride efflux and mitochondrial dysfunction. 2GBI treatment alone promoted anti-tumor immunity and inhibited tumor growth via NLRP3-dependent manner. Moreover, 2GBI treatment could overcome ICB resistance and exerted synergistic anti-tumor effects. These results suggest that targeting NLRP3 is a potential strategy to induce anti-tumor immunity and improve the efficacy of ICB.

## Introduction

The immune checkpoint blockade (ICB) has shown great clinical efficacy in cancer therapy [1–3]. However, tumors with low T cell infiltration, so called “cold tumors”, are resistant to the ICB treatment [4]. Recent studies show that the activation of innate immunity can induce T cell infiltration in tumor microenvironment and enhance the sensitivity to ICB to inhibit tumor growth [5–7]. The agonists of innate immune receptors are currently in development as both monotherapy and in combination with ICB. For example, treatment with agonists of TLR7, TLR9, or STING has been reported to enhance the anti-tumor activity and overcome ICB resistance in mouse models [8–10]. However, the efficacy and prospects of these drugs in clinical therapy are still unclear. Therefore, it is of great importance to develop new strategies targeting innate immunity for the treatment of tumors.

As an important innate immune receptor, NLRP3 responds to multiple kinds of stimuli and mediates assembly of the inflammasome complex, leading to production of inflammatory cytokines and pyroptosis [11–14]. Recent studies demonstrated that the NLRP3 inflammasome activation induces IL-1β-dependent priming of T cells to upregulate cytotoxicity in diverse tumor models, indicating that NLRP3 is a potential target for immunotherapy [15]. Several molecules that induce NLRP3 inflammasome activation have shown anti-tumor activity in mouse models and clinical studies. Treatment with NLRP3 agonist BMS-392959 results in significant tumor control in murine tumor models, but whether the therapeutic effect of BMS-392959 is dependent on NLRP3 is unknown [16]. NLRP3 agonist BMS-986299 in combination with anti-PD-1 shows modest clinical activity in advanced solid tumors in a phase I clinical study. However, whether BMS-986299 is able to enhance therapeutic effects of anti-PD-1 and whether BMS-986299 inhibits tumor progression through acting on NLRP3 need further investigation [17]. VTX-2337 has been reported to induce NLRP3 activation, and shows anti-tumor activity in combination with cetuximab in SCCHN patients. However, VTX-2337 can also initiate TLR8 activation, and there is no evidence that VTX-2337 inhibits tumorigenesis through activating NLRP3 [18, 19]. Thus, whether targeting NLRP3 is an effective strategy to enhance anti-tumor immunity and overcome ICB resistance needs to be studied.

Here, we found 2GBI as an agonist directly targeting NLRP3. 2GBI is a selective and state-dependent blocker of voltage-gated proton channels (Hv1) and has not been reported to play a role in inflammatory response yet [20]. Our results showed that 2GBI induces inflammasome assembly and activation by binding to NLRP3 and promoted anti-tumor immunity in a NLRP3-dependent manner. In addition, 2GBI treatment could overcome the resistance to anti-PD-1 therapy in cold tumors.

## Results

### 2GBI induces caspase-1 maturation and IL-1β production in macrophages

**Fig. 1:**
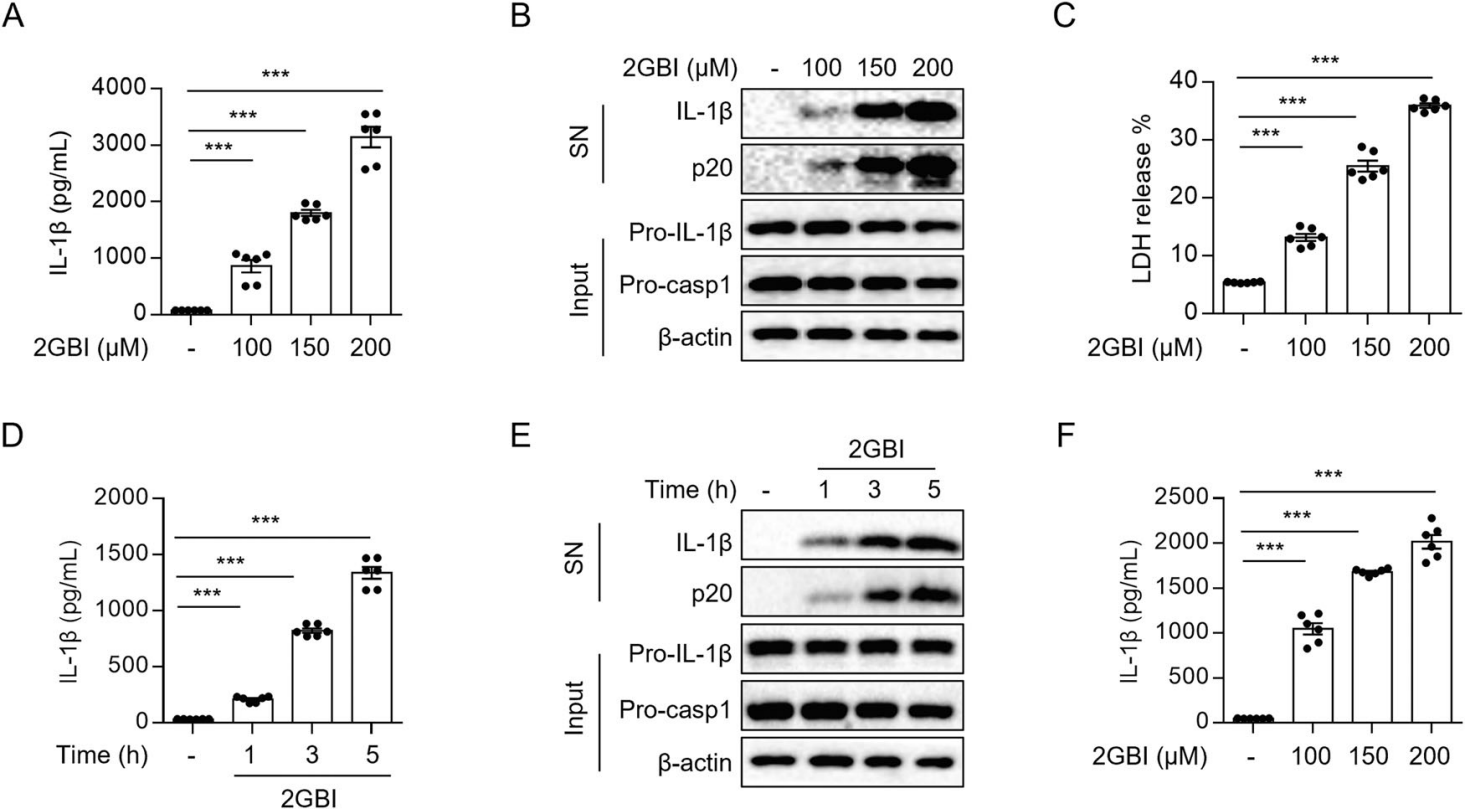
2GBI induces caspase-1 maturation and IL-1β release. **A**–**C** LPS-pretreated BMDMs were stimulated with different concentrations of 2GBI (100 μM, 150 μM, 200 μM) for 40 min. **A** ELISA analysis of IL-1β in the supernatants (*n* = 6). **B** Western blot analysis of IL-1β and p20 (mature caspase-1) in the supernatants (SN) and pro-IL-1β and pro-caspase-1 in the lysates (Input). **C** LDH release in the SN (*n* = 6). **D**, **E** LPS-pretreated BMDMs were stimulated with 100 μM 2GBI for 1 h, 3 h, or 5 h. **D** ELISA analysis of IL-1β in the SN (*n* = 6). **E** Western blot analysis of IL-1β and p20 in the SN and pro-IL-1β and pro-caspase-1 in the Input. **F** LPS-pretreated PBMCs were stimulated with different concentrations of 2GBI (100 μM, 150 μM, 200 μM) for 40 min. The level of IL-1β in the SN was analyzed by ELISA (*n* = 6). Data are derived from three independent experiments (**A**, **C**, **D**, **F**) and displayed by mean ± SEM or represent three independent experiments (**B**, **E**). Statistical significance was analyzed by unpaired Student’s t-test: ***P < 0.001.

To provide a potential approach for the treatment of tumor, we found a small molecule named 2GBI, a Hv1 channel blocker, that could induce IL-1β secretion in BMDMs. Then, we treated LPS-primed BMDMs with 2GBI, and detected the protein levels in cell supernatants and lysates by ELISA and western blot. We found that 2GBI could induce caspase-1 cleavage and IL-1β secretion in both dose- and time-dependent manner, and also dose-dependently induced IL-18 release (Fig. 1A, B, D, E, Supplementary Fig. 1A, B). In addition, 2GBI dose-dependently promoted the release of lactate dehydrogenase (LDH) from BMDMs (Fig. 1C). We stimulated LPS-primed human peripheral blood mononuclear cells (PBMCs) with 2GBI and found that 2GBI could also induce IL-1β release in a dose-dependent manner in human cells (Fig. 1F). In addition, our data showed that 2GBI did not induce IL-6 or TNF-α production (Supplementary Fig. 2A, B), nor did it affect IL-6 or TNF-α production in LPS-primed BMDMs (Supplementary Fig. 2D, E). 2GBI did not induce the activation of MAPK signaling pathway (Supplementary Fig. 2C). In summary, these results indicate that 2GBI can induce caspase-1 maturation and IL-1β secretion in macrophages.

### 2GBI induces NLRP3 inflammasome activation

**Fig. 2:**
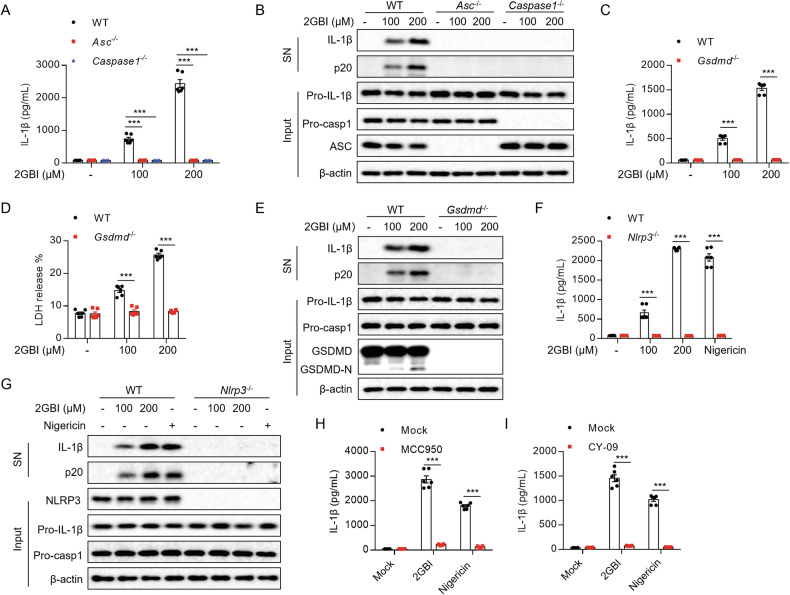
2GBI activates NLRP3 inflammasome. **A**, **B** WT, *Asc*^*−/−*^ or *Caspase-1*^*−/−*^ BMDMs were pretreated with LPS for 3 h, and then stimulated with 2GBI (100 μM, 200 μM) for 40 min. **A** ELISA analysis of IL-1β in the SN (*n* = 6). **B** Western blot analysis of IL-1β and p20 in the SN and pro-IL-1β, pro-caspase-1, and ASC in the Input. **C**–**E** WT or *Gsdmd*^*−/−*^ BMDMs were pretreated with LPS for 3 h, and then stimulated with 2GBI (100 μM, 200 μM) for 40 min. **C** ELISA analysis of IL-1β in the SN (*n* = 6). **D** LDH release in the SN (*n* = 6). **E** Western blot analysis of IL-1β and p20 in the SN and pro-IL-1β, pro-caspase-1, and GSDMD in the Input. **F**, **G** WT or *Nlrp3*^*−/−*^ BMDMs were pretreated with LPS for 3 h, and then stimulated with 2GBI (100 μM, 200 μM) or nigericin (3 μM). **F** ELISA analysis of IL-1β in the SN (*n* = 6). **G** Western blot analysis of IL-1β and p20 in the SN and pro-IL-1β, pro-caspase-1, and NLRP3 in the Input. **H**, **I** LPS-pretreated BMDMs were treated with MCC950 (200 nM) or CY-09 (20 μM) for 30 min and then stimulated with 2GBI (200 μM) or nigericin (3 μM). The level of IL-1β in the SN was analyzed by ELISA (*n* = 6). Data are derived from three independent experiments (**A**, **C**, **D**, **F**, **H**, **I**) and displayed by mean ± SEM or represent three independent experiments (**B**, **E**, **G**). Statistical significance was analyzed by unpaired Student’s t-test: ***P < 0.001.

The inflammasome complex is composed of pattern recognition receptor, ASC, and caspase-1 [21]. The assembly of inflammasome induces caspase-1 maturation, IL-1β release, and pyroptosis [14]. We isolated BMDMs from WT (Wild-type), *Asc*^*−/−*^ and *Caspase-1*^*−/−*^ mice, treated these cells with LPS and stimulated with 2GBI. We found that 2GBI failed to induce the release of mature caspase-1 and IL-1β in *Asc*^*−/−*^ or *Caspase-1*^*−/−*^ BMDMs (Fig. 2A, B). During the activation of inflammasome, GSDMD mediates pyroptosis and promotes the release of inflammatory factors [22]. To detect whether 2GBI-mediated IL-1β and p20 secretion was dependent on GSDMD, we used 2GBI to stimulate WT and *Gsdmd*^*−/−*^ BMDMs. Our data showed that IL-1β and caspase-1 maturation and LDH release induced by 2GBI were significantly inhibited in *Gsdmd*^*−/−*^ BMDMs (Fig. 2C–E).

A variety of pattern recognition receptors, such as NLRP3, AIM2, Pyrin, and NLRC4, can form inflammasomes with ASC and caspase-1 [23]. Firstly, we treated LPS-primed WT and *Nlrp3*^*−/−*^ BMDMs with 2GBI and found that 2GBI induced IL-1β secretion and caspase-1 cleavage in a NLRP3-dependent manner (Fig. 2F, G). In addition, we investigated whether NLRP3 inhibitors MCC950 [24] and CY09 [25] affected the activation of inflammasome by 2GBI. As expected, both MCC950 and CY-09 significantly inhibited 2GBI-induced IL-1β release (Fig. 2H, I). These results indicate that 2GBI induces NLRP3 inflammasome activation. Furthermore, we used 2GBI to stimulate WT and *Caspase11*^*−/−*^ BMDMs and detected the IL-1β secretion. The inflammasome activation induced by 2GBI was independent of caspase11, so 2GBI could not induce the non-canonical inflammasome activation (Supplementary Fig. 3A). We next treated LPS-primed WT, *Aim2*^*−/−*^, *Mefv* (*Pyrin*)^*−/−*^ and *Nlrc4*^*−/−*^ BMDMs with 2GBI. We found that 2GBI activated inflammasome independently of AIM2, Pyrin, or NLRC4 (Supplementary Fig. 3B–G). The results indicate that 2GBI specifically activates the NLRP3 inflammasome.

### 2GBI-mediated NLRP3 activation is independent of upstream events and HVCN1

**Fig. 3:**
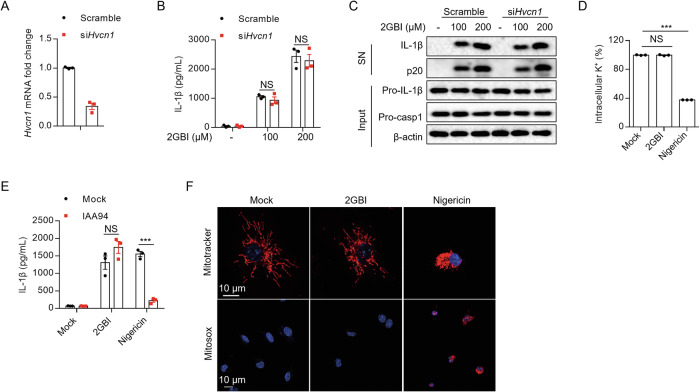
2GBI-mediated NLRP3 activation is independent of upstream events and HVCN1. **A**–**C***Hvcn1* gene in BMDMs was knocked down by siRNA. Next, the cells were pretreated with LPS for 3 h, and then stimulated with 2GBI (100 μM, 200 μM) for 40 min. **A** qPCR analysis of *Hvcn1* mRNA expression (*n* = 3). **B** ELISA analysis of IL-1β in the SN (*n* = 3). **C** Western blot analysis of IL-1β and p20 in the SN and pro-IL-1β and pro-caspase-1 in the Input. **D** Intracellular potassium concentration of LPS-pretreated BMDMs stimulated with 2GBI (200 μM) or nigericin (3 μM) (*n* = 3). **E** LPS-pretreated BMDMs were stimulated with IAA94 (100 μM) for 30 min and then stimulated with 2GBI (200 μM) or nigericin (3 μM). The level of IL-1β in the SN of BMDMs was analyzed by ELISA (*n* = 3). **F** Confocal microscopy analysis of LPS-pretreated BMDMs stimulated with 2GBI (200 μM) or nigericin (3 μM) and then stained with Mitotracker (red), Mitosox (red), and DAIP (blue). Data are derived from three independent experiments (**A**, **B**, **D**, **E**) and displayed by mean ± SEM or represent three independent experiments (**C**, **F**). Statistical significance was analyzed by unpaired Student’s *t*-test: ***P < 0.001, NS no significance.

Next, we explored the mechanism of 2GBI-mediated NLRP3 inflammasome activation. 2GBI has been regarded as an inhibitor of the voltage-gated proton channel HVCN1, through binding to the VSD domain [20]. To investigate whether 2GBI-mediated NLRP3 activation is regulated by HVCN1, we knocked down *Hvcn1* with siRNA in BMDMs and found that 2GBI-induced IL-1β release was not affected (Fig. 3A–C). The results indicate that HVCN1 has no effects on 2GBI-induced NLRP3 inflammasome activation.

Potassium efflux, chloride efflux, mitochondrial damage, and reactive oxygen species production have been reported to be important upstream events of NLRP3 inflammasome activation [21, 26, 27]. By detecting the intracellular potassium concentration in BMDMs after stimulation, we found that 2GBI did not affect the cellular potassium level, while nigericin significantly reduced cellular potassium concentration (Fig. 3D). In addition, we replaced the culture supernatants of LPS-primed BMDMs with different concentrations of potassium medium, and found that inhibiting potassium efflux did not affect 2GBI-induced IL-1β secretion (Supplementary Fig. 4A). These results suggest that 2GBI activates NLRP3 inflammasome independently of potassium efflux. Then, we used IAA94, an intracellular chloride channel inhibitor [28], to study whether chloride flux affected the activation of NLRP3 inflammasome induced by 2GBI. IAA94 did not inhibit IL-1β release induced by 2GBI (Fig. 3E). Meanwhile, we used MQAE to evaluate the level of cellular chloride and found that 2GBI stimulation did not decrease the concentration of cellular chloride (Supplementary Fig. 4B), indicating that 2GBI activates NLRP3 inflammasome independently of chloride efflux. Finally, we examined the effects of 2GBI on mitochondrial damage and ROS production. After 2GBI treatment, the cells were stained with Mitotracker or Mitosox and analyzed for confocal microscopy. We found that 2GBI could not cause mitochondrial damage or ROS production in BMDMs (Fig. 3F). We also observed the same results by flow cytometry analysis (Supplementary Fig. 4C–F). These results suggest that 2GBI does not cause mitochondrial damage and reactive oxygen species production during activation of NLRP3 inflammasome. In summary, the activation of NLRP3 inflammasome induced by 2GBI is independent of the upstream signaling events and HVCN1.

### 2GBI binds to NLRP3 and promotes inflammasome assembly

**Fig. 4:**
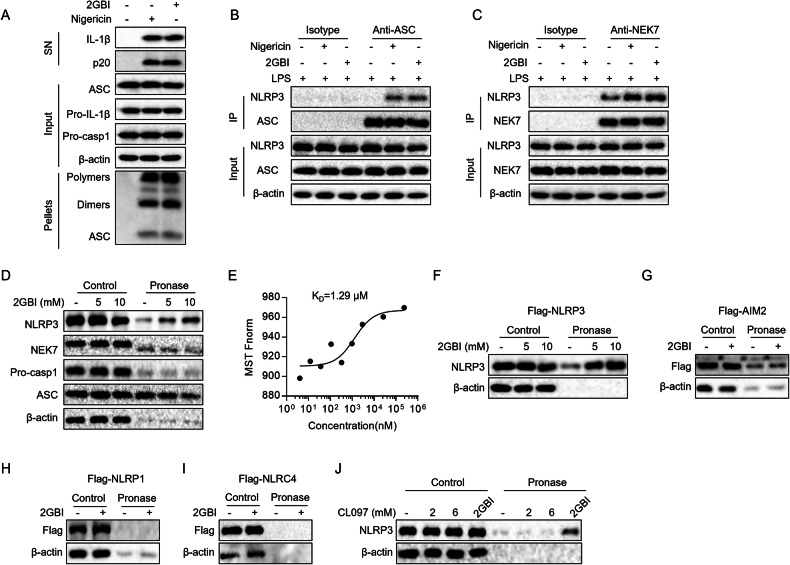
2GBI binds NLRP3 and promotes inflammasome assembly. **A** Western blot analysis of ASC oligomerization in TBS-insoluble pellets of LPS-pretreated BMDMs stimulated with 2GBI (200 μM) or nigericin (3 μM). Endogenous immunoprecipitation and western blot to detect the NLRP3-ASC interaction (**B**) and NLRP3-NEK7 interaction (**C**) in LPS-pretreated BMDMs stimulated with 2GBI (200 μM) or nigericin (3 μM). **D** The lysates of LPS-pretreated BMDMs were incubated with different concentrations of 2GBI (5 mM, 10 mM) overnight and then digested with pronase (25 ng/μg of protein). NLRP3, NEK7, pro-caspase-1, and ASC in lysates were analyzed by western blot. **E** MST analysis to detect the affinity between 2GBI and GFP-NLRP3 protein. The lysates of HEK-293T cells transfected with flag-tagged NLRP3, AIM2, NLRP1, or NLRC4 plasmids were incubated with 2GBI overnight and then digested with pronase (25 ng/μg of protein). Flag-NLRP3 (**F**), flag-AIM2 (**G**), flag-NLRP1 (**H**), or flag-NLRC4 (**I**) in lysates were analyzed by western blot. **J** The lysates of HEK-293T cells transfected with flag-NLRP3 plasmid were incubated with CL097 (2 mM, 6 mM) or 2GBI (10 mM) overnight and then digested with pronase (25 ng/μg of protein). The flag-NLRP3 in lysates was analyzed by western blot. All data represent three independent experiments.

To investigate the mechanism of 2GBI-induced NLRP3 activation, we next detected whether 2GBI interacted with the components of NLRP3 inflammasome. ASC oligomerization is an important step in the assembly of NLRP3 inflammasome and triggers subsequent cleavage of caspase-1 [29]. To detect ASC oligomerization, we performed cross-linking experiments using dextran sulfate sodium (DSS) and found that 2GBI efficiently induced ASC oligomerization in BMDMs (Fig. 4A). The interaction between NLRP3 and ASC is a prerequisite for the formation of ASC oligomers [29]. Co-immunoprecipitation assay showed that 2GBI promoted strong endogenous NLRP3-ASC interaction in BMDMs (Fig. 4B). NEK7 is a serine/threonine protein kinase that interacts with NLRP3 to induce NLRP3 oligomerization [30]. 2GBI could also induce NLRP3-NEK7 interaction, which was proved by co-immunoprecipitation experiments (Fig. 4C). Taken together, these results suggest that 2GBI is able to promote NLRP3 inflammasome assembly.

To explore how 2GBI promotes NLRP3 inflammasome assembly, we detected the interaction between 2GBI and NLRP3 protein through drug affinity response target stability (DARTS) assay and microthermophoresis (MST). The mechanism of DARTS assay is that the proteins binding with molecules are protected from degradation by pronase, thus the resistance to degradation indicates the interaction between molecules and proteins [31]. We added 2GBI and pronase to the lysates of LPS-primed BMDMs, and found that 2GBI could dose-dependently reduce the degradation of NLRP3 rather than NEK7, ASC, or caspase-1 (Fig. 4D). Additionally, we co-incubated the purified His-GFP-NLRP3 protein with 2GBI to perform the MST test. MST results showed that the K_D_ value between 2GBI and His-GFP-NLRP3 was 1.29 μM (Fig. 4E), indicating that 2GBI has a high affinity with His-GFP-NLRP3. The above results prove that 2GBI can bind to NLRP3.

To explore whether 2GBI binds to other pattern recognition receptors, we overexpressed NLRP3 or other inflammasome sensors in HEK-293T cells and performed DARTS experiments, and found that 2GBI bound to NLRP3 rather than AIM2, NLRP1, or NLRC4 (Fig. 4F-I). CL097 is a NLRP3 agonist that mediates NLRP3 activation through inhibiting mitochondrial complex I to induce ROS production, and has no effects on intracellular potassium level [32]. Although both CL097 and 2GBI do not induce intracellular potassium efflux, CL097 cannot bind to NLRP3 (Fig. 4J). Collectively, 2GBI is a NLRP3 agonist which specifically binds to NLRP3. NLRP3 is composed of PYD, NACHT, and LRR domains [21]. We found that 2GBI interacted with the LRR domain of NLRP3, but not PYD or NACHT, indicating that 2GBI may induce NLRP3 activation through altering the cage structure (Supplementary Fig. 5A–C).

### 2GBI promotes anti-tumor immunity and overcome ICB resistance

**Fig. 5:**
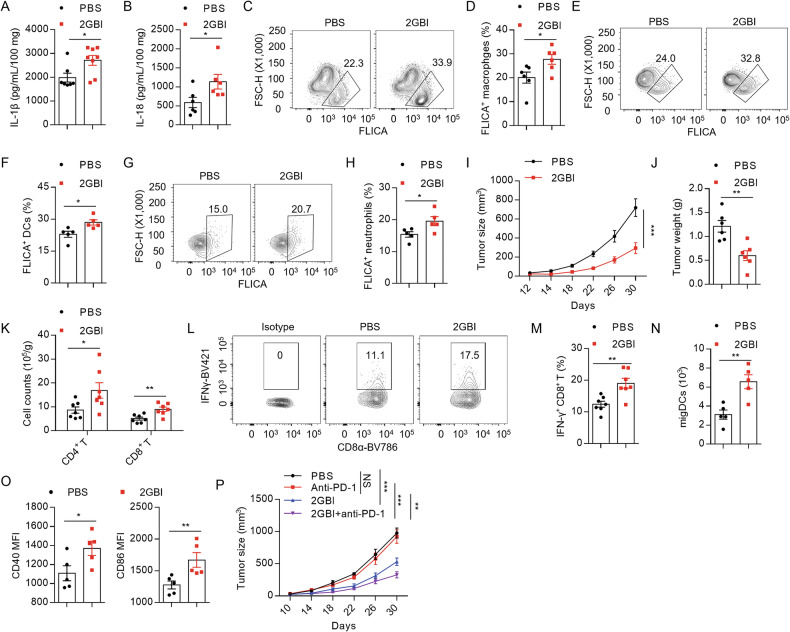
2GBI promotes anti-tumor immunity and overcome ICB resistance. **A**–**H**, **N**, **O** Mice subcutaneously implanted with LLC cells were intraperitoneally injected with 2GBI (20 mg/kg) or PBS vehicle every two days. Mice were sacrificed after three times of administration. ELISA analysis of IL-1β (**A**) (*n* = 7, 8) and IL-18 (**B**) (*n* = 6) in the supernatants from tumor tissues after culture for 24 h. Flow cytometry analysis of caspase-1^+^ macrophages (**C**), caspase-1^+^ DCs (**E**), and caspase-1^+^ neutrophils (**G**) from tumor tissues. Quantitative analysis of caspase-1^+^ macrophages (**D**) (*n* = 6), caspase-1^+^ DCs (**F**) (*n* = 5), and caspase-1^+^ neutrophils (**H**) (*n* = 5) from tumor tissues. **I**–**M** Mice subcutaneously implanted with LLC cells were intraperitoneally injected with 2GBI (20 mg/kg) or PBS vehicle every two days. Mice were sacrificed 30 days after tumor cells implantation. LLC tumor growth curve (**I**) and weight (**J**) (*n* = 6). **K** Quantitative analysis of tumor-infiltrating CD4^+^ T and CD8^+^ T cells (*n* = 7). Flow cytometry analysis (**L**) and quantification (**M**) of IFN-γ^+^ CD8^+^ T cells (*n* = 7). **N** Quantitative analysis of migratory DCs from inguinal lymph nodes (*n* = 5). **O** Mean fluorescence intensities (MFIs) of CD40 and CD86 in DCs from inguinal lymph nodes (*n* = 5). **P** LLC tumor growth in mice treated with PBS, anti-PD-1, 2GBI, or 2GBI plus anti-PD-1 (*n* = 5, 6). Data represent two (**A**, **B**, **D**, **F**, **H**, **N**–**P**) or three (**I**–**K**) independent experiments or are derived from two independent experiments (**M**). All Data are displayed by mean ± SEM. Statistical significance was analyzed by unpaired Student’s t-test or two-way ANOVA for (**I**, **P**): *P < 0.05, **P < 0.01, ***P < 0.001, NS no significance.

To investigate the role of NLRP3 agonist in antitumor immunity, mice with subcutaneously implanted LLC tumors were intraperitoneally injected with 20 mg/kg 2GBI or PBS vehicle every other day. The results showed that the inflammasome was activated in tumors (Fig. 5A, B), which may be induced by danger signals from dead or stressed cells in tumor microenvironment. 2GBI treatment increased the levels of IL-1β and IL-18 and the proportion of caspase-1^+^ macrophages in tumor tissues (Fig. 5A–D), indicating that 2GBI can induce inflammasome activation in tumors. In addition to macrophages, 2GBI promoted the inflammasome activation in neutrophils and DCs in tumors (Fig. 5E–H). To examine whether 2GBI can induce inflammasome activation in DCs in vitro, we stimulated LPS-primed BMDCs with 2GBI and found that 2GBI stimulation could induce IL-1β secretion in DCs in vitro (Supplementary Fig. 6A, B).

2GBI significantly inhibited the growth of LLC and B16F10 tumors (Fig. 5I, J and Supplementary Fig. 7A, B). In the LLC tumor model, 2GBI treatment increased the number of tumor-infiltrating CD4^+^ T and CD8^+^ T cells (Fig. 5K), and the percentage of IFN-γ^+^ CD8^+^ T cells (Fig. 5L, M) in the tumor, indicating that 2GBI enhances the anti-tumor activity of T cells. In tumor-draining lymph nodes, 2GBI treatment increased infiltration of DCs and promoted activation of DCs, leading to stronger anti-tumor response (Fig. 5N, O). In the spleen of tumor-burdened mice, 2GBI treatment increased the proportion of T cells and NK cells but did not affect the level of myeloid cells (Supplementary Fig. 8A–G). Furthermore, intratumoral injection of 2GBI achieve better inhibitory effects of tumor growth than intraperitoneal injection in LLC tumor models (Supplementary Fig. 9A, B). To test the safety of the application, we observed that dosing lower than 40 mg/kg 2GBI did not induce acute inflammation, and repeated dosing of 20 mg/kg 2GBI showed no significant toxicity in mice (Supplementary Fig. 10A–F).

In cold tumors, the therapeutic effects of ICB are limited because of low targeting cell infiltration. The activation of innate immunity can induce T cell infiltration in tumor microenvironment in order to enhance the sensitivity to ICB. Considering the activator role of 2GBI in innate immune response, 2GBI may enhance T cell infiltration and activation and then change cold tumors into warm tumors via inducing activation of tumor-infiltrated macrophage and DCs. To investigate the effect of 2GBI in ICB therapy, 2GBI and PBS injected tumor-bearing mice received anti-PD-1 or control treatment. While the LLC tumor growth was resistant to anti-PD-1 therapy, 2GBI treatment could inhibit LLC growth. Furthermore, 2GBI treatment significantly enhances the therapeutic effects of anti-PD1 antibody in LLC tumor-burdened mice (Fig. 5P). Therefore, 2GBI is a potent immune stimulator, which can be used as an immunotherapy agent in combination with ICB to improve the anti-tumor response and benefit patients barely respond to ICB treatment. Collectively, 2GBI can efficiently initiate anti-tumor immunity and overcomes ICB resistance.

### 2GBI promotes anti-tumor immunity via NLRP3-dependent manner

**Fig. 6:**
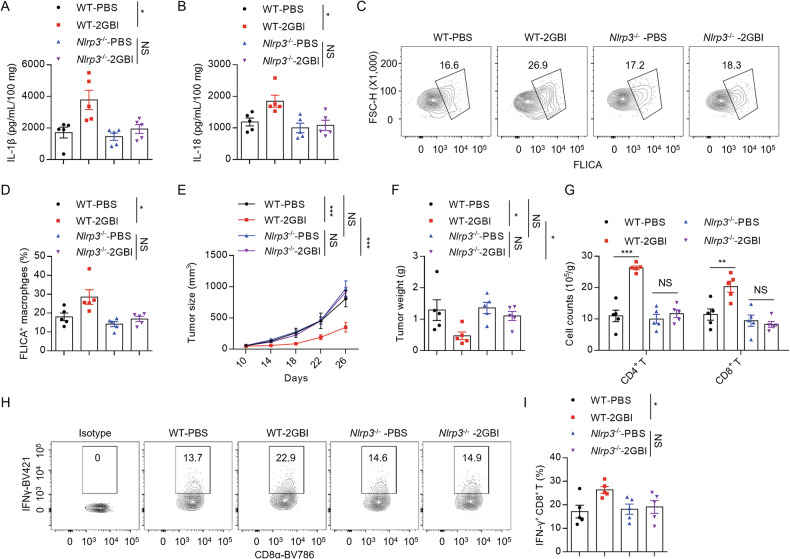
2GBI promotes antitumor immunity via NLRP3-depedent manner. **A**–**D** WT or *Nlrp3*^*−/−*^ mice subcutaneously implanted with LLC cells were intraperitoneally injected with 2GBI (20 mg/kg) or PBS vehicle every two days. Mice were sacrificed after three times of administration. ELISA analysis of IL-1β (**A**) and IL-18 (**B**) in the supernatants of tumor tissues after culture for 24 h (*n* = 5). Flow cytometry analysis (**C**) and quantification (**D**) of caspase-1^+^ macrophages (*n* = 5) from tumor tissues. **E**–**I** WT or *Nlrp3*^*−/−*^ mice subcutaneously implanted with LLC cells were intraperitoneally injected with 2GBI (20 mg/kg) or PBS vehicle every two days. Mice were sacrificed 26 days after tumor cells implantation. Tumor growth curve (**E**) and weight (**F**) (*n* = 5). **G** Quantification of tumor infiltrating CD4^+^ T and CD8^+^ T cells (*n* = 5). Flow cytometry analysis (**H**) and quantification (**I**) of IFN-γ^+^ CD8^+^ T cells (*n* = 5). Data represent two (**A**, **B**, **D**, **G**, **I**) or three (**E**, **F**) independent experiments. All Data are displayed by mean ± SEM. Statistical significance was analyzed by unpaired Student’s t-test or two-way ANOVA for (**E**): *P < 0.05, **P < 0.01, ***P < 0.001, NS no significance.

Next, we studied whether the anti-tumor activity of 2GBI depends on NLRP3. WT or *Nlrp3*^*−/−*^ mice with subcutaneously implanted LLC cells were intraperitoneally injected with 2GBI or PBS. The results showed that the levels of IL-1β and IL-18 in tumor tissues induced by 2GBI were significantly reduced in *Nlrp3*^*−/−*^ mice (Fig. 6A, B), and 2GBI promoted the increase of caspase-1^+^ macrophage in a NLRP3-dependent manner (Fig. 6C, D), suggesting that 2GBI induces activation of NLRP3 inflammasome in tumors. In addition, 2GBI could not inhibit tumor growth and promote tumor cytotoxic T cell infiltration and activation in *Nlrp3*^*−/−*^ mice (Fig. 6E–I). These results suggest that 2GBI promotes anti-tumor immunity in an NLRP3-dependent manner.

## Discussion

In this study, we demonstrate that 2GBI is a novel NLRP3 agonist which directly binds to NLRP3 and promotes NLRP3-mediated immune response in tumor. Furthermore, we show that treatment of 2GBI to trigger NLRP3 activation can overcomes the resistance of ICB in cold tumors. These results suggest that NLRP3 is an effective target to enhance anti-tumor activity, and provide a candidate strategy for tumor immunotherapy via triggering NLRP3.

The immune checkpoint blocking therapy targeting PD-1/PD-L1 has made great progress in a variety of tumors [33–35]. However, this strategy is not suitable for the treatment of cold cancers with low immune cell-infiltration [36]. Our study provides a new solution to enhance the sensitivity of ICB therapy in cold tumors. While the single anti-PD-1 treatment poorly inhibited the growth of LLC tumors, anti-PD-1 combination with NLRP3 agonist 2GBI could significantly inhibit LLC tumor growth. NLRP3 activation has been reported to play controversial roles in tumor microenvironment. In dendritic cells, the NLRP3 inflammasome activation and IL-1β secretion promote the priming of CD8^+^ T cells and T cell-mediated killing of tumor cells [37]. In macrophages, the NLRP3 activation suppresses Th1 cell polarization and CD8^+^ T cell activation [38]. Thus, the mechanisms for 2GBI-mediated tumor killing need further investigation.

The agonists of innate immune receptors are currently in development as new tumor therapy strategies. However, substantial barriers remain for the clinical use of targeting these pathways. Treatment of some agonist induces pathologic inflammation, due to the over-activation of innate immune sensors. For example, squamous cell carcinoma of head and neck (SCCHN) patients received TLR9 agonist IMO-2055 experience sepsis, bacteremia, and hyperthermia [39]. Treatment of TLR7 agonist LHC165 is associated with risk of neutropenia, lymphopenia, and pancreatitis in patients with advanced solid tumors [40]. Our results show that intraperitoneally injection of 20 mg/kg 2GBI is effective to inhibit tumor growth and overcome ICB resistance, without inducing systemic inflammation, indicating that 2GBI could be safe and tolerable. Since improvement of tumor microenvironment by 2GBI could enhance the responsiveness of cold tumors to ICB treatment, 2GBI or its analog has great potential to become an adjuvant in tumor therapy. However, considering that the current activity of 2GBI is relatively low, it is necessary to identify its binding site on NLRP3 by using structural biology or other methods and optimize its activity in the future.

In conclusion, this study provides a new NLRP3 activator and reveals that activating NLRP3 can induce potent antitumor immunity. These results also suggest that NLRP3 agonist provides an opportunity to overcome ICB resistance.

## Materials and methods

### Mice

C57BL/6J mice were purchased from the Shanghai SLAC Laboratory Animal Ltd. Corp. (Shanghai, China). *Nlrp3*^*−/−*^ mice were provided by Dr. Jurg Tschopp’s group. *Asc*^*−/−*^ and *Nlrc4*^*−/−*^ mice were provided by the group of Dr. Vishva M. Dixit. *Capsase-1*^*−/−*^ mice were provided by the group of Dr. Richard A. Flavell. *Gsdmd*^*−/−*^ mice were provided by Dr. Shu Zhu’s group. *Aim2*^*−/−*^ mice were provided by Dr. Bing Sun’s group. *Mevf*^*−/−*^ mice were provided by Dr. Feng Shao’s group. *Capsase11*^*−/−*^ mice were provided by the group of Dr Qingde Wang. Sex and age-matched mice were specific pathogen-free (SPF) and housed in a strict 12 h light/12 h dark cycle (lights on at 08: 00 and off at 20:00) at 22–24 °C with unrestricted food and water.

### Reagents

2GBI (G11802) was purchased from Sigma-Aldrich. Mitotracker, Mitosox, DAPI, Pam3CSK4, MQAE, Lipofectamine RNAiMAX, and Lipofextamine 2000 were from Invitrogen. Mouse Flt-3 Ligand (FLT3L) recombinant protein, Mitotracker green, and Mitotracker deep red were obtained from Thermo Fisher Scientific. LPS, nigericin, poly (dA:dT,) and IAA94 were bought from Sigma-Aldrich. C3 toxin was synthesized by Tengchuan Jin’s group. The Salmonella strain (*Salmonella typhimurium*) was a gift from Dr. Cai Zhang’s group. CY-09 and MCC950 were purchased from Selleck. CL097 was from InvivoGen. Protein G Agarose (16-266) was bought from Millipore. Protein Ladder (26616) was bought from Thermo Fisher Scientific. Protein Ladder (WJ103) was bought from Yazyme Biotech. Anti-Flag beads (A2220) and anti-Flag antibody (F2555) were purchased from Sigma-Aldrich. Anti-NLRP3 antibody (AG-20B-0014) and anti-caspase-1 antibody (AG-20B-0042) were bought from AdipoGen. Anti-mouse IL-1β antibody (AF-401-NA) was purchased from R&D Systems. Anti-NEK7 antibody (ab133514) was from Abcam. Anti-ASC antibody (67824S), anti-IL-18 antibody (57058), phospho-p44/42 MAPK (Erk1/2) (Thr202/Tyr204) (E10) antibody (9106), phospho-SAPK/JNK (Thr183/Tyr185) antibody (9251), phospho-p38 MAPK (Thr180/Tyr182) antibody (9211), phospho-IκBα (Ser32/36) (5A5) antibody (9246) and mouse anti-rabbit IgG (Conformation Specific) (L27A9) antibody (5127) were purchased from Cell Signaling Technology. Anti-β-actin antibody (66009-1-Ig) was purchased from Proteintech. Anti-PD-1 antibody (BP0146) was purchased from Bio X Cell.

### Cell culture and stimulation

All cells were cultured in an incubator containing 5% CO_2_ at 37 °C. Bone marrow was isolated from adult mice and cultured in Dulbecco modified Eagle Medium (DMEM) containing 10% FBS, 1% antibiotics, and 20 ng/mL M-CSF (Novoprotein) for 4–5 days to differentiate into bone marrow-derived macrophages (BMDMs). Bone marrow cells were cultured in Roswell Park Memorial Institute (RPIM)-1640 containing 10% FBS, 1% antibiotics, and 100 ng/mL FLT3L for 15 days to induce bone marrow-derived DCs (BMDCs). Culture of LLC lung carcinoma cells and B16F10 melanoma cells have been described previously [41]. All of the cell lines have been tested for mycoplasma contamination. Human peripheral blood mononuclear cells (PBMCs) from the peripheral blood of healthy people were obtained using the Human Lymphocyte Separation Medium kit (Solarbio), and then cultured in Roswell Park Memorial Institute (RPIM)-1640 containing 10% FBS and 1% antibiotics.

Digest differentiated BMDMs with 5 mM EDTA, and plate 5 × 10^5^/mL BMDMs in 12-well plates overnight. Next, cells were primed with 50 ng/mL LPS (for canonical inflammasome activation) or 400 ng/mL Pam3CSK4 (for non-canonical inflammasome activation) for 3 h. Afterwards, cells were stimulated with 2GBI, nigericin (3 μM) for 20 min, C3 toxin (1 μg/mL) for 6 h, or *S. typhimurium* for 2 h. Cells were transfected with poly (dA:dT) (1 μg/mL) for 2 h or LPS (500 ng/mL) for 16 h using Lipofectamine 2000. For inhibition experiments of NLRP3 inflammasome activation, cells were treated with MCC950 (200 nM), CY09 (20 μM), or IAA94 (100 μM) for 30 min before stimulation with 2GBI or nigericin. The lactate dehydrogenase (LDH) detection experiment was completed according to the instructions of the lactate dehydrogenase cytotoxicity detection kit (Beyotime). The cell supernatants were used to detect inflammatory factors by ELISA kit. Cell lysates and supernatants were collected for western blot.

5 × 10^5^/mL BMDCs or PBMCs were placed in 12-well plates overnight. The next morning the culture medium was changed to OPTI-MEM containing 1% FBS and 50 ng/mL LPS for 3 h, and then the cells were stimulated with different concentrations of 2GBI for 40 min.

### ELISA

Supernatants of BMDMs and tumor tissues were tested for mouse IL-1β (R&D, DY-401) and IL-18 (Invitrogen, BMS618-3). The supernatants of BMDMs were also tested for mouse IL-6 (R&D, DY406) and TNF-α (R&D, DY410). The supernatants of BMDCs and peritoneal lavage fluid were tested for mouse IL-1β. The supernatants of PBMCs were tested for human IL-1β (R&D, 557953). All experiments were performed according to the instructions of the ELISA kit.

### Western blot

The protein in the sample buffer was boiled at 101 °C for 10 min, and the electrophoresis experiment was carried out. The conditions of gel running were 80 V for 30 min in concentrated gel and 120 V for 60 min in separation gel. Then, transfer the proteins in the gel to a PVDF membrane in transfer buffer at 90 V for 60 min. The PVDF membrane was incubated in blocking solution for 30 min and then incubated with primary antibody overnight at 4 °C on a shaker. The next day, the PVDF membrane was washed three times with PBST and incubated with secondary antibodies for 1 h at room temperature. The PVDF membrane was washed three times with PBST. The membrane reacted with the HRP substrate and was then exposed in a chemiluminescence gel imager to obtain an image.

### Confocal microscopy

2 × 10^5^/mL BMDMs were placed on 24-well plates overnight. The next morning, the NLRP3 inflammasome activation experiments were performed. Cells were stained with Mitotracker (50 nM) and Mitosox (5 μM) for 30 min before the end of stimulation. The supernatants were removed and the cells were washed three times with PBS. Cells were fixed with 4% paraformaldehyde at room temperature for 15 min, and then washed three times with PBST for 5 min each time. The nuclei were stained with DAIP. Finally, the cells were observed by Zeiss LSM 700.

### Silencing genes in BMDMs by siRNA

2.5 × 10^5^/mL cells were plated in 12-well plates overnight. Then, the supernatants were changed to OPTI-MEM, and the cells were transfected with siRNA (50 nM) for 6 h using lipoRNAiMAX. Then, the cells were cultured in DMEM containing 10% FBS, 1% antibiotics, and 20 ng/mL M-CSF at 37 °C. After 48 h, the gene knockdown effect was detected and the NLRP3 inflammasome activation experiment was performed. *siHvcn1* sequence was CCACAGGTTTCAGGTCATCAT.

### Quantitative real-time PCR

BMDMs were lysed with TRIzol reagent (Tsingke), and then RNA was extracted using chloroform and isopropanol. One micogram RNA was reverse transcribed into cDNA using the M-MLV Reverse Transcriptase kit (Invitrogen) according to the manufacturer’s instructions. cDNA was used as template for quantitative PCR using SYBR Green premix (Abclonal). *Gapdh* was used as a reference gene. The primer sequences were as follows:

*mHvcn1*-forward: 5′-CACTTTACGGTGGTTGGGGA-3′;

*mHvcn1*-reverse: 5′-AGCTCATGTAGTGGAACGCC-3′;

*mGapdh*-forward: 5′-GGTGAAGGTCGGTGTGAACG-3′;

*mGapdh*-reverse: 5′-CTCGCTCCTGGAAGATGGTG-3′.

### Detection of intracellular potassium and chloride concentrations

Place of 1 × 10^6^/mL BMDMs in 6-well plates. NLRP3 inflammasome activation experiments were performed, and then the supernatants were removed. The cells were washed three times with K^+^-free PBS and then lysed with nitric acid. The cell lysates were transferred to a beaker and boiled dry until light yellow powder appeared. The powder was dissolved with 2 mL ddH_2_O, and then the concentration of potassium in the solution was detected by inductively coupled plasma emission spectroscopy.

To measure the intracellular chloride concentration, 5 × 10^5^/mL BMDMs were plated in 12-well plates overnight. NLRP3 inflammasome activation experiments were performed. Then, the supernatants were removed and the cells were lysed with ddH_2_O for 15 min at 37 °C. The lysates were transferred to a 1.5 mL centrifuge tube and placed for 30 min at −80 °C, followed by centrifugation at 8000 rpm for 5 min. Fifty microliter supernatant was mixed with 50 μL MQAE (10 μM), and the absorbance of the mixture was measured in a BioTek Multi-Mode Microplate Reader (Synergy2).

### ASC oligomerization detection

BMDMs (1 × 10^6^/mL) were placed in 6-well plates for NLRP3 inflammasome activation. Cells were lysed with TBS buffer for 30 min at 4 °C on a shaker. Cell lysates were centrifuged for 15 min at 6000 × *g*/4 °C. The pellets were washed 3 times with TBS for 1 min each time at 6000 × *g*/4 °C, and resuspended in 500 uL TBS containing 2 mM DSS. The resuspended pellets were cross-linked for 30 min at 37 °C and inverted every 15 min. Afterwards, the samples were centrifuged for 15 min at 6000 × *g*/4 °C. The supernatants were removed and the pellets were resuspended in 50 μL 3× sample buffer. Finally, the samples were analyzed by western blot.

### Endogenous co-immunoprecipitation assay

After activating the NLRP3 inflammasome, BMDMs (1 × 10^6^/mL) in 6-well plates were lysed with NP-40 containing protease inhibitors for 30 min at 4 °C on a shaker. The cell lysates were centrifuged to remove impurities, and then Protein G Mag Sepharose was added to the cell lysates. The primary antibody or IgG was co-incubated with the cell lysates at 4 °C overnight. The next day, the samples were washed several times with NP-40 without protease inhibitors and centrifuged. The pellets were resuspended with 50 μL 3× sample buffer and the results were presented by western blot.

### Drug Affinity Responsive Target Stability (DARTS) assay

HEK-293T cells were transfected with plasmids for 24 h or BMDMs were stimulated with LPS for 3 h in a 10 cm culture dish. Cells were lysed by NP-40 containing protease inhibitors for 30 min at 4 °C on a shaker. Remove impurities from the cell lysates by centrifugation. The protein concentration in the lysates was detected by a Pierce BCA Protein Assay Kit (Biosharp). Incubate lysates with small molecules overnight at 4 °C in a rotating dish. Then, pronase (25 ng/μg of protein, Sigma–Aldrich) was added to the lysates and incubated at room temperature for 30 min. Subsequently, 3× sample buffer was added to the cell lysates to stop the reaction. The samples were analyzed by western blot.

### Protein expression and purification

Expression and purification of His-GFP-NLRP3 were performed as previously described [42].

### Microscale thermophoresis (MST) assay

Three-fold serial dilutions of 2GBI (0.238 mM to 4.03 nM) were incubated with 20 μg purified His-GFP-NLRP3 protein at room temperature for 30 min in the test buffer (50 mM Hepes, 10 mM MgCl_2_, 100 mM NaCl, 0.05% Tween 20, and pH 7.5). The samples were then placed in glass capillaries for MST analysis. MST detection was performed under 80% MST power and 100% LED power. The results were analyzed by Monolith NT.115 instrument and K_D_ values were calculated using Graphpad Software.

### Plasmid constructions

PCR reaction was performed by PrimeSTAR® Max DNA polymerase (TaKaRa). PCR product recovery was performed by SanPrep Column Plasmid Mini-Preps kit (Sangon Biotech). Gene recombination was performed by ClonExpress® II one-step cloning kit (Vazyme). The plasmid was finally determined by sequencing.

### Histology

Tissues were fixed in 4% PFA overnight and then paraffin-embedded, sectioned, and stained with hematoxylin and eosin (H&E).

### 2GBI-induced acute inflammation in vivo

Eight-week-old sex and age-matched mice were randomized and intraperitoneally injected with different concentrations of 2GBI. Six hours later, the mice were sacrificed. The peritoneal cavities were flushed with 1 mL PBS, and the peritoneal lavage fluids were centrifuged. The supernatants were used to detect IL-1β. Then, the peritoneal cavities were flushed twice with 10 mL PBS. The peritoneal lavage fluids were centrifuged and the supernatants were removed. The precipitates of the above three peritoneal lavage fluids were mixed to obtain peritoneal lavage fluid cells.

### Tumor colonization and in vivo treatment

Eight- to twelve-week-old sex and age-matched mice were randomized and subcutaneously injected with 1.5 × 10^6^ LLC cells or 4 × 10^5^ B16F10 cells. Tumor size was monitored using vernier calipers. The volume of the tumor was calculated using volume = length × width × width × 0.5.

For the 2GBI treatment, after LLC transplantation for 10–12 days or B16F10 transplantation for 7 days, the mice were randomly assigned to receive intraperitoneal injections (IP) or intratumoral injections (IT) of PBS vehicle or 20 mg/kg 2GBI every other day. For the anti-PD-1 treatment, On the 10th, 13th, and 17th days after LLC transplantation, the mice were treated with 200 ug/per mouse anti-PD-1 antibody.

### Cell collection

To obtain tumor-infiltrating lymphocytes, mice were euthanized. Carefully dissect tumors from mice. Tumor tissues were cut into small pieces using scissor instruments and then digested in RPIM-1640 containing 1 mg/mL collagenase VI (Sigma-Aldrich) and 0.1 mg/mL DNase I (Sigma-Aldrich) for 30 min at 37 °C on a shaker. Digested tumor tissues were centrifuged. Then, the pellets were resuspended in 40% percoll solution and density gradient centrifuged. Remove the supernatants and resuspend the pellets in PBS to obtain cell suspensions. Finally, filter the cell suspensions through a 70 μm cell strainer to obtain single cells. To obtain lymphocytes from tumor-draining lymph nodes, mice were euthanized after three treatments. The inguinal lymph nodes were minced with scissors and then digested in RPIM-1640 containing 1 mg/mL collagenase VI and 0.1 mg/mL DNase I at 37 °C on a shaker for 30 min. The digested lymph node tissues were centrifuged and single-cell suspensions were obtained through a cell strainer. Spleens were ground through a 40 μm cell strainer, and then red blood cells were removed by RBC lysis buffer (Biosharp).

### Flow cytometry analysis

BMDMs (3 × 10^5^/well) were placed in a low-adsorption 96-well plate (LABSELECT) and NLRP3 inflammasome activation experiments were performed. Cells were stained with Mitosox (5 μM) or Mitotracker green (50 nM) and Mitotracker deep red (50 nM) for 30 min, and were then washed with PBS containing 1% FBS and analyzed by FACS.

Single cell suspensions from tumor tissues were stained with fluorescence-conjugated antibodies. For cytokine staining, cells were stimulated for 3 h with 50 ng/mL PMA (Sigma–Aldrich), 1 μg/mL ionomycin (Sigma–Aldrich), and 2.5 μg/mL monensin (Sigma–Aldrich) at 37 °C in RPIM-1640. Cells were stained with surface antibody for 30 min at 4 °C, and then fixed for 40 min at room temperature with Fixation/Permeabilization buffer and stained with intracellular antibodies. The cells were resuspended in PBS solution and analyzed by flow cytometry. For the detection of caspase-1 activation in tumor tissues, cells were stained with FAM-FLICA® Caspase-1 (YVAD) Assay Kit (Immunochemistry Technologies) for FACS analysis.

### Statistical analysis

No data were excluded from the analyzes. Sample sizes were selected on the basis of preliminary results to ensure adequate power. The histology analysis was conducted in a blinded fashion. Data were analyzed by two-sided unpaired t-test or two-way ANOVA (Graphpad software) and expressed as mean ± SEM. The variance between the groups that are being statistically compared is similar. Differences between data were considered significant when *P* < 0.05.

## Supplementary information

Supplementary Figures

Original data

## Data Availability

The data supporting the findings of this study are available from the corresponding author upon reasonable request.
