# Supplementary material for: A small molecule directly targets NLRP3 to promote inflammasome activation and antitumor immunity
Source: Cell Death Dis. 2025 Apr 4;16(1):252. doi: 10.1038/s41419-025-07578-0 (PMC11971322; doi:10.1038/s41419-025-07578-0)
Supplement: Supplementary file 1 — Supplementary Figures [file 41419_2025_7578_MOESM1_ESM.docx]

**Supplementary Fig. 1**

**
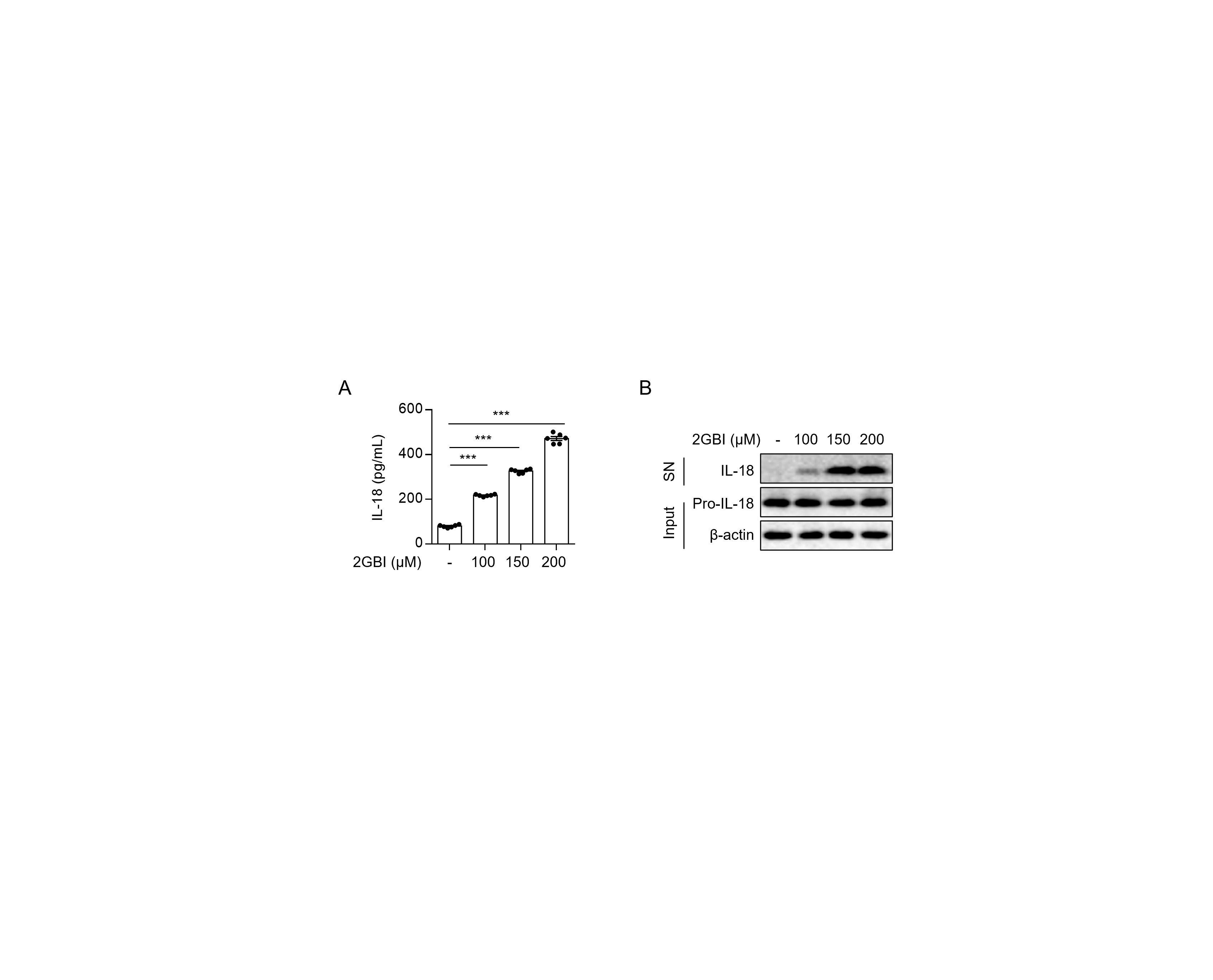
**

**Supplementary Fig. 1** **2GBI induces IL-18 maturation and release,** **related to Fig.1. (A, B)** LPS-pretreated BMDMs were stimulated with different concentrations of 2GBI (100 μM, 150 μM, 200 μM) for 40 min. **(A)** ELISA analysis of IL-18 in the SN (n = 6). **(B)** Western blot analysis of IL-18 in the SN and pro-IL-18 in the Input. Data are derived from three independent experiments **(A)** and displayed by mean ± SEM or represent three independent experiments **(B)**. Statistical significance was analyzed by unpaired Student 's t-test: ****P* < 0.001.

**Supplementary Fig. 2**

**
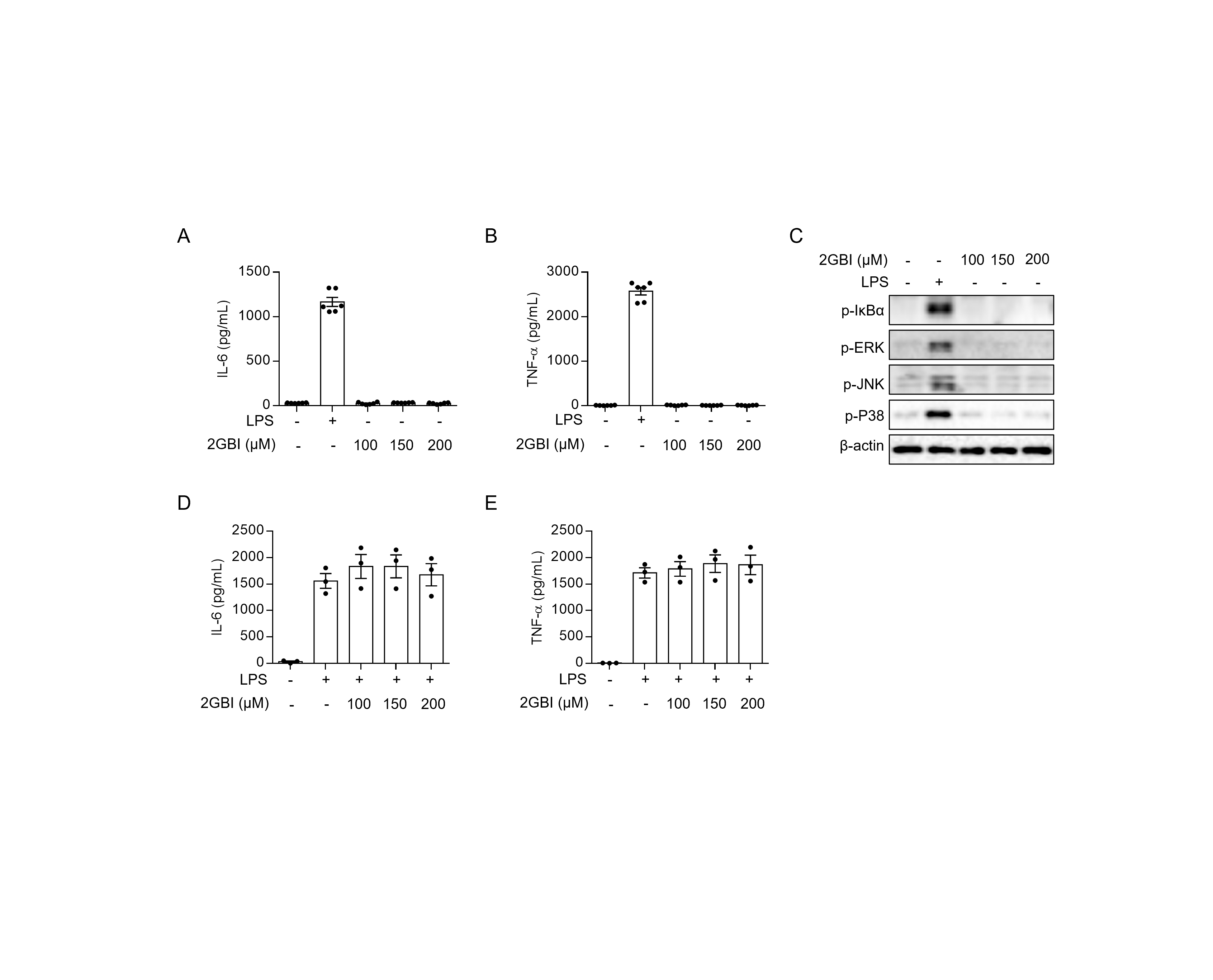
**

**Supplementary Fig. 2** **2GBI does not affect the LPS-priming signal, related to Fig.1. (A-C)** BMDMs were stimulated with LPS (50 ng/mL) or different concentrations of 2GBI (100 μM, 150 μM, 200 μM) for 3 h. ELISA analysis of IL-6 **(A)** (n = 6) and TNF-α **(B)** (n = 6) in the SN. **(C)** Western blot analysis of p-IκBα, p-ERK, p-JNK and p-P38 in the Input. **(D, E)** LPS-primed BMDMs were stimulated with 2GBI (100 μM, 150 μM, 200 μM) for 40 min. ELISA analysis of IL-6 **(D)** (n = 3) and TNF-α **(E)** (n = 3) in the SN. Data are derived from three independent experiments **(A, B, D, E)** or represent three independent experiments **(C)**.

**Supplementary Fig. 3**


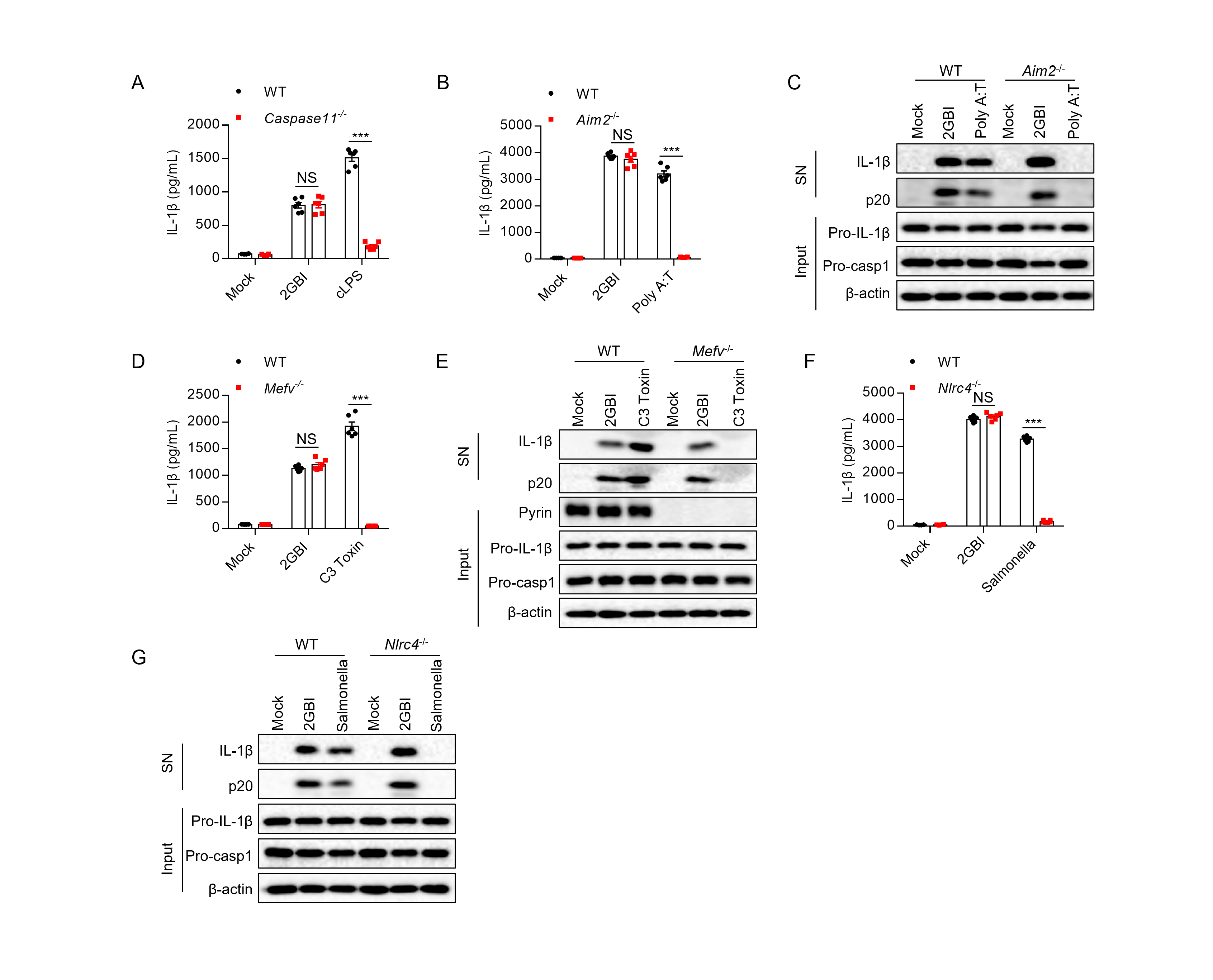


**Supplementary Fig. 3** **2GBI activates inflammasome independently of caspase11, AIM2, Pyrin or NLRC4, related to Fig.2. (A)** WT or *Caspase11^-/-^* BMDMs were primed with Pam3CSK4 for 3 h, and then stimulated with 2GBI (200 μM ) or cytosolic LPS (cLPS) (500 ng/mL). ELISA analysis of IL-1β in the SN (n = 6). **(B, C)** WT or *Aim2^-/-^* BMDMs were pretreated with LPS for 3 h, and then stimulated with 2GBI (200 μM ) or poly (dA:dT) (1 μg/mL). **(B)** ELISA analysis of IL-1β in the SN (n = 6). **(C)** Western blot analysis of IL-1β and p20 in the SN and pro-IL-1β and pro-caspase-1 in the Input. **(D, E)** WT or *Mefv^-/-^* BMDMs were pretreated with LPS for 3 h, and then stimulated with 2GBI (200 μM) or C3 toxin (1 μg/mL). **(D)** ELISA analysis of IL-1β in the SN (n = 6). **(E)** Western blot analysis of IL-1β and p20 in the SN and pro-IL-1β, pro-caspase-1 and pyrin in the Input. **(F, G)** WT and *Nlrc4^-/-^* BMDMs were pretreated with LPS for 3 h, and then stimulated with 2GBI (200 μM) or *Salmonella* (10 MOI). **(F)** ELISA analysis of IL-1β in the SN (n = 6). **(G)** Western blot analysis of IL-1β and p20 in the SN and pro-IL-1β and pro-caspase-1 in the Input. Data are derived from three independent experiments **(A, B, D, F)** and displayed by mean ± SEM or represent three independent experiments **(C, E, G)**. Statistical significance was analyzed by unpaired Student 's t-test: ****P* < 0.001, NS, no significance.

**Supplementary Fig. 4**

**
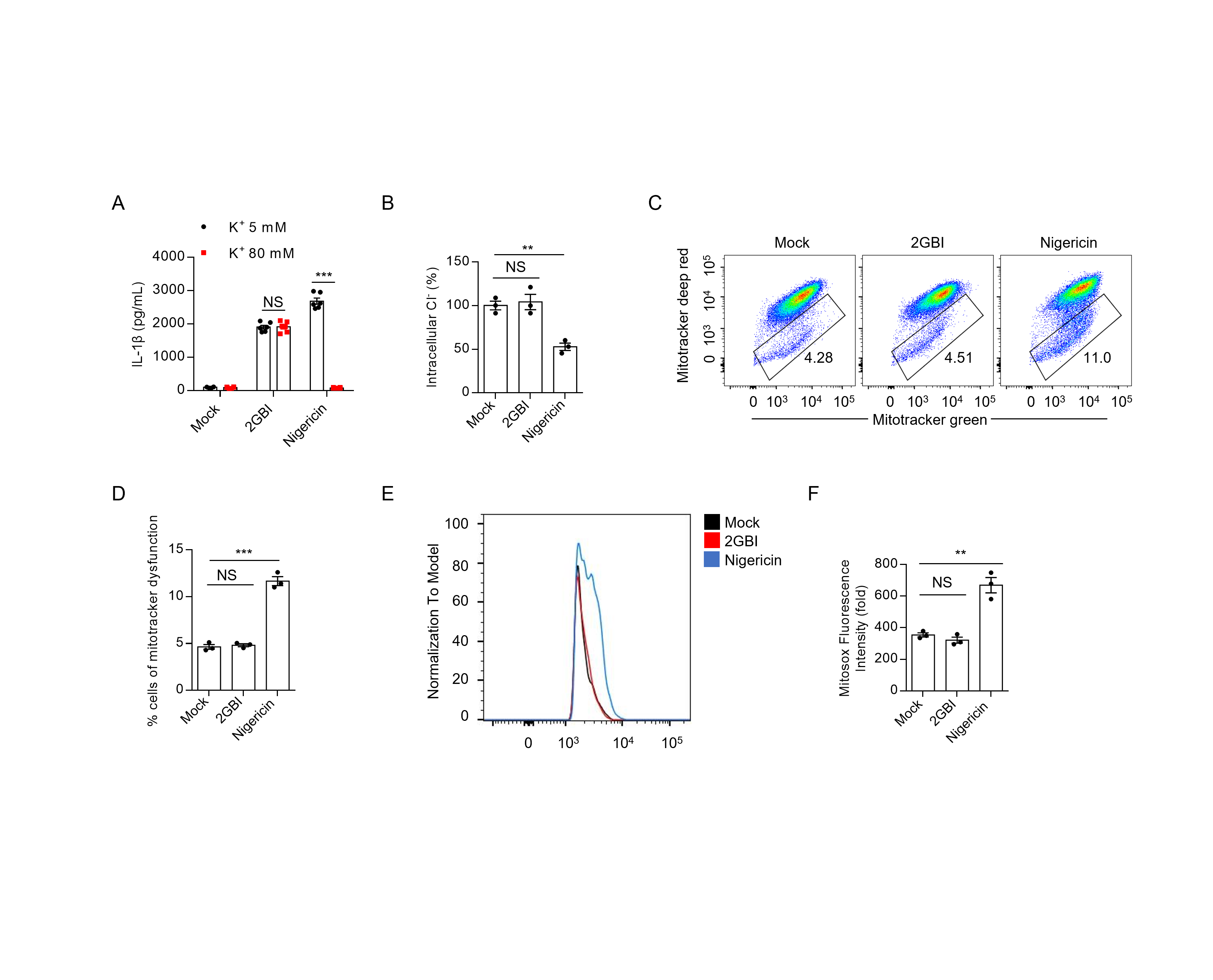
**

**Supplementary Fig. 4** **2GBI activates NLRP3 inflammasome independently of potassium ion efflux,** **chloride ion efflux, mitochondrial damage, or ROS production, related to Fig.3. (A)** The supernatants of LPS-pretreated BMDMs were replaced with medium containing different concentrations of potassium (5 mM, 80 mM), and then the cells were stimulated with 2GBI (200 μM) or nigericin (3 μM). The level of IL-1β in the SN was analyzed by ELISA (n = 6). **(B)** Intracellular chloride ion concentration of LPS-pretreated BMDMs stimulated with 2GBI (200 μM) or nigericin (3 μM) (n = 3). Flow cytometry analysis **(C)** and quantification **(D)** of LPS-pretreated BMDMs stimulated with 2GBI (200 μM) or nigericin (3 μM) and then stained with Mitotracker deep red and Mitotracker green (n = 3). Flow cytometry analysis **(E)** and quantification **(F)** of LPS-pretreated BMDMs stimulated with 2GBI (200 μM) or nigericin (3 μM) and stained with Mitosox (n = 3). Data are derived from three independent experiments **(A, B, D, F)** and displayed by mean ± SEM or represent three independent experiments **(C, E)**. Statistical significance was analyzed by unpaired Student 's t-test: ***P* < 0.01, ****P* < 0.001, NS, no significance.

**Supplementary Fig. 5**

**
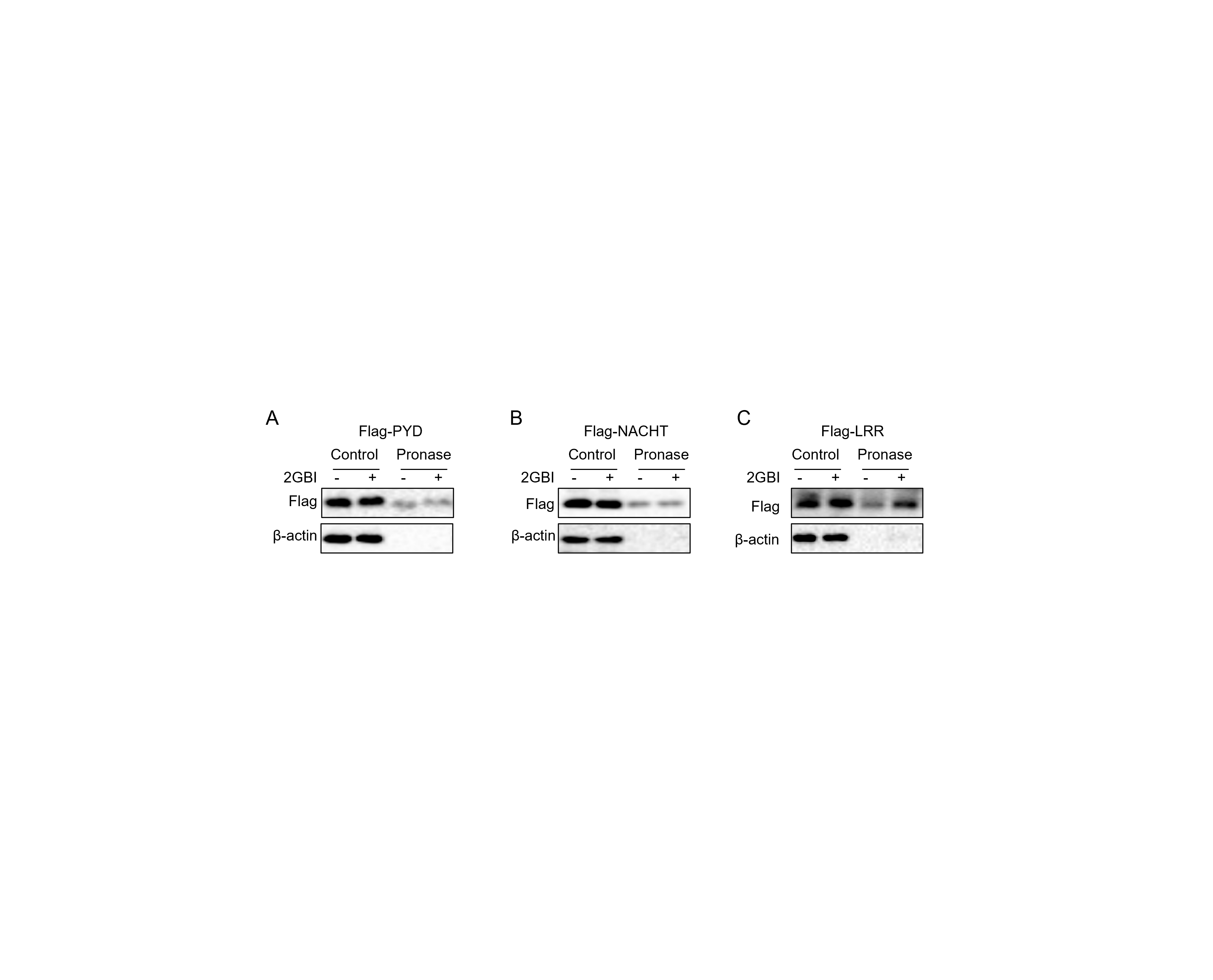
**

**Supplementary Fig. 5. 2GBI binds to the LRR domain of NLRP3, related to Fig.4.** The lysates of HEK-293T cells transfected with flag-tagged PYD, NACHT, or LRR plasmids were incubated with 2GBI overnight and then digested with pronase (25 ng/μg of protein). Flag-PYD **(A)**, flag-NACHT **(B)**, or flag-LRR **(C)** in lysates were analyzed by western blot. All data represent three independent experiments.

**Supplementary Fig. 6**

**
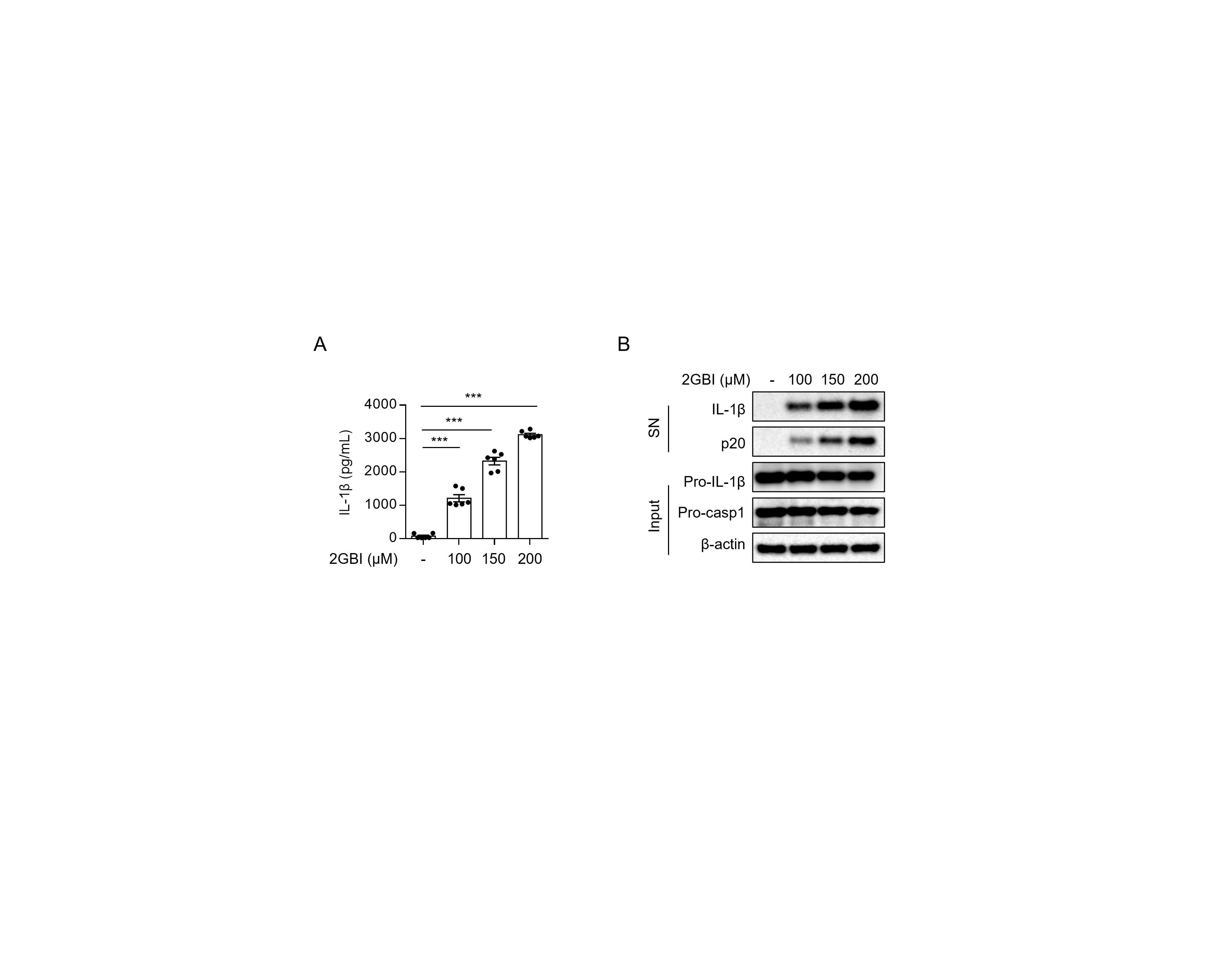
**

**Supplementary Fig. 6. 2GBI activates NLRP3 inflammasome in BMDCs, related to Fig.5. (A, B)** LPS-pretreated BMDCs were stimulated with different concentrations of 2GBI (100 μM, 150 μM, 200 μM) for 40 min. **(A)** ELISA analysis of IL-β in the SN (n = 6). **(B)** Western blot analysis of IL-1β and p20 in the SN and pro-IL-1β and pro-caspase-1 in the Input. Data are derived from three independent experiments **(A)** and displayed by mean ± SEM or represent three independent experiments **(B)**. Statistical significance was analyzed by unpaired Student 's t-test: ****P* < 0.001.

**Supplementary Fig. 7**

**
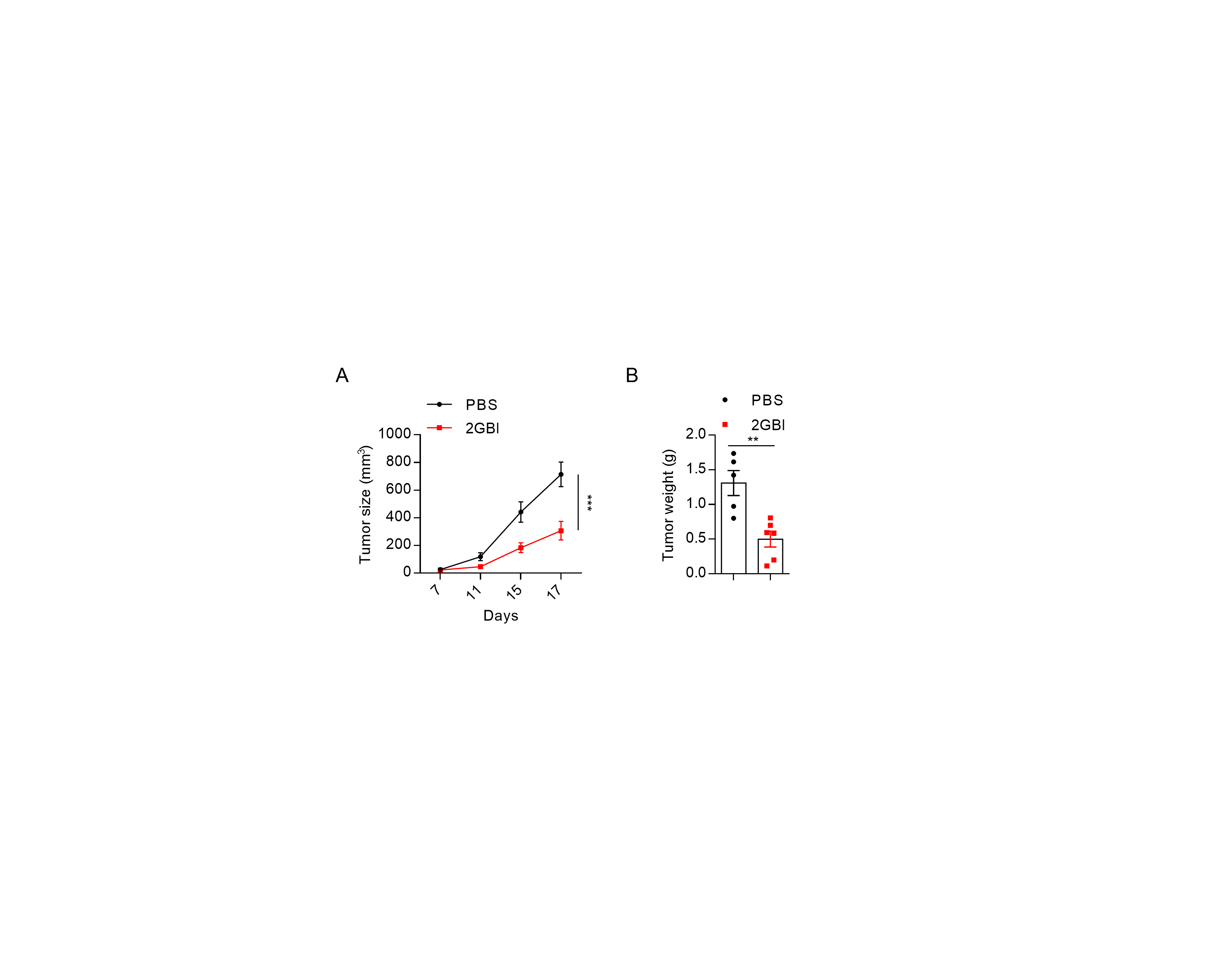
**

**Supplementary Fig. 7.** **2GBI inhibits the growth of B16F10 tumors, related to Fig.5. (A, B)** Mice subcutaneously implanted with B16F10 cells were intraperitoneally injected with 2GBI (20 mg/kg) or PBS vehicle every two days. Mice were sacrificed 17 days after tumor cell implantation. B16F10 tumor growth curve **(A)** and weight **(B)** (n = 5, 6). Data represent two independent experiments and are displayed by mean ± SEM. Statistical significance was analyzed by unpaired Student 's t-test for **(B)** or two-way ANOVA for **(A)**: ***P* < 0.01, ****P* < 0.001.

**Supplementary Fig. 8**

**
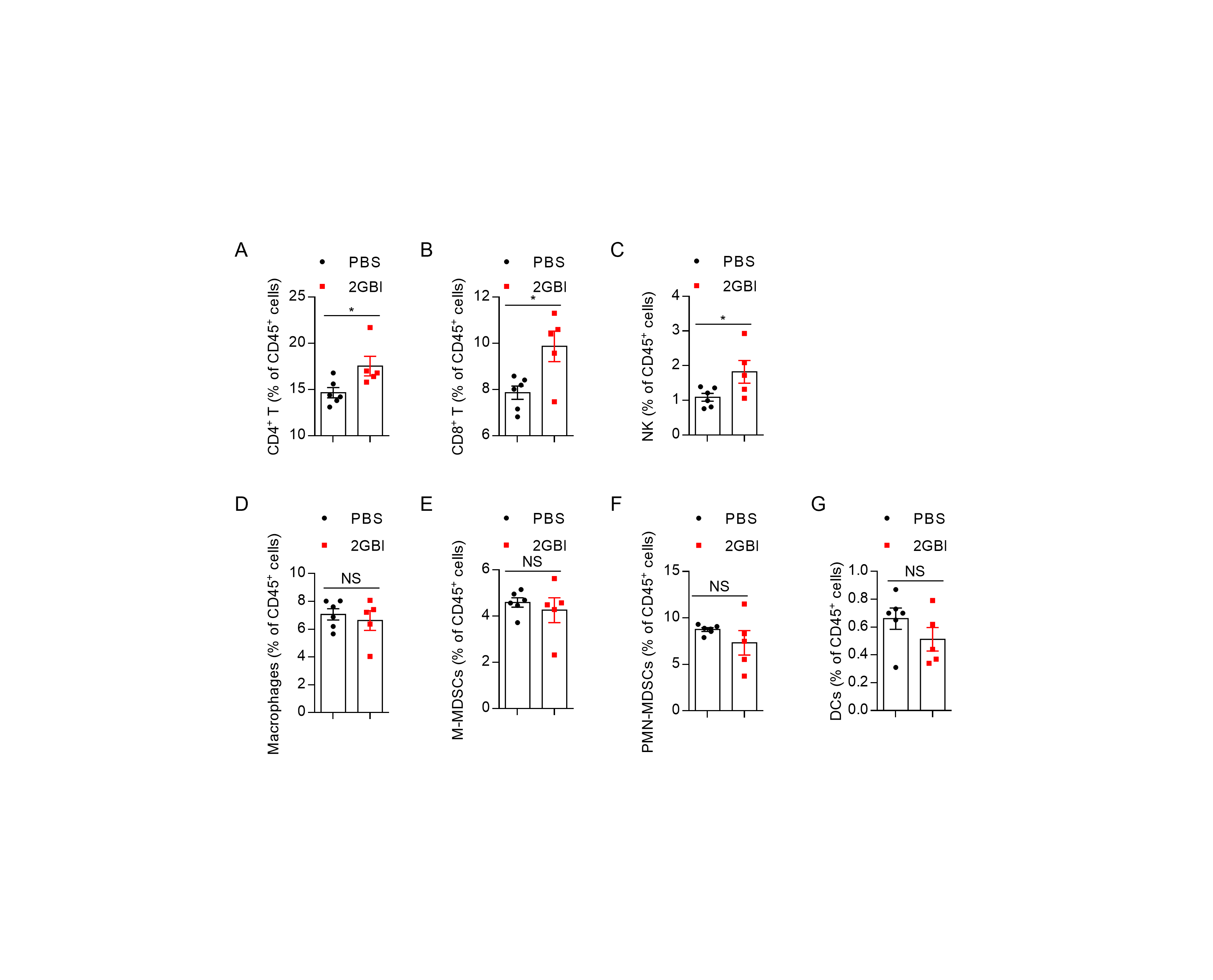
**

**Supplementary Fig. 8. 2GBI increases the proportion of cytotoxic cells from spleens, related to Fig.5. (A-G)** Mice subcutaneously implanted with LLC cells were intraperitoneally injected with 2GBI (20 mg/kg) or PBS vehicle every two days. Mice were sacrificed 30 days after tumor cells implantation. Quantitative analysis of CD4^+^ T cells **(A)**, CD8^+^ T cells **(B)**, NK cells **(C)**, macrophages **(D)**, M-MDSCs **(E)**, PMN-MDSCs **(F)** and DCs **(G)** from spleens (n = 5, 6). Data represent two independent experiments and are displayed by mean ± SEM. Statistical significance was analyzed by unpaired Student 's t-test: **P* < 0.1, NS, no significance.

**Supplementary Fig. 9**


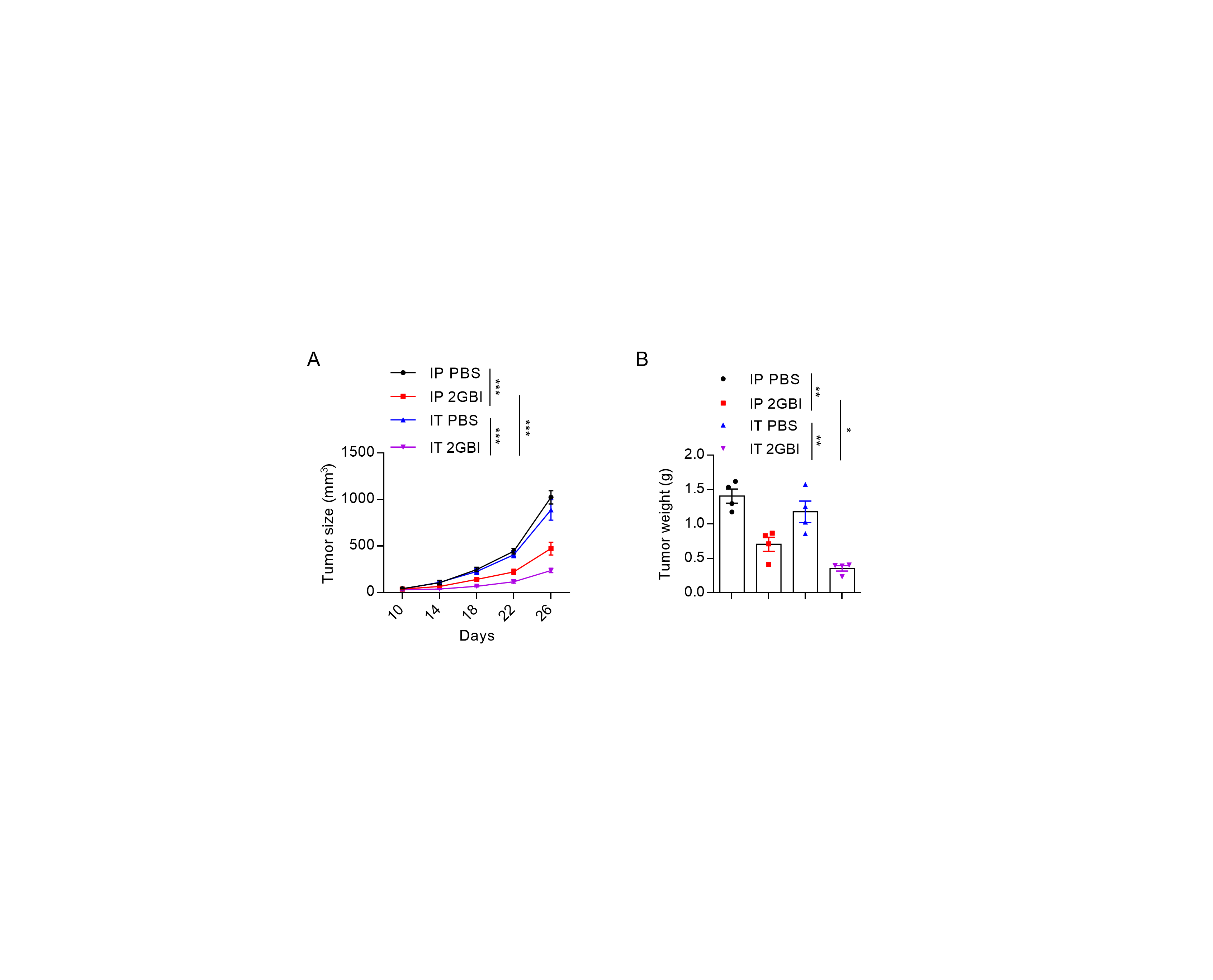


**Supplementary Fig. 9. Intratumoral injection of 2GBI is more effective than intraperitoneal injection in inhibiting tumor growth, related to Fig.5. (A, B)** Mice subcutaneously implanted with LLC cells were intraperitoneally injected (IP) or intratumorally injected (IT) with 2GBI (20 mg/kg) or PBS vehicle every two days. Mice were sacrificed 26 days after tumor cells implantation. LLC tumor growth curve **(A)** and weight **(B)** (n = 4). Data represent two independent experiments and are displayed by mean ± SEM. Statistical significance was analyzed by unpaired Student 's t-test for **(B)** or two-way ANOVA for **(A)**: **P* < 0.1, ***P* < 0.01, ****P* < 0.001.

**Supplementary Fig. 10**


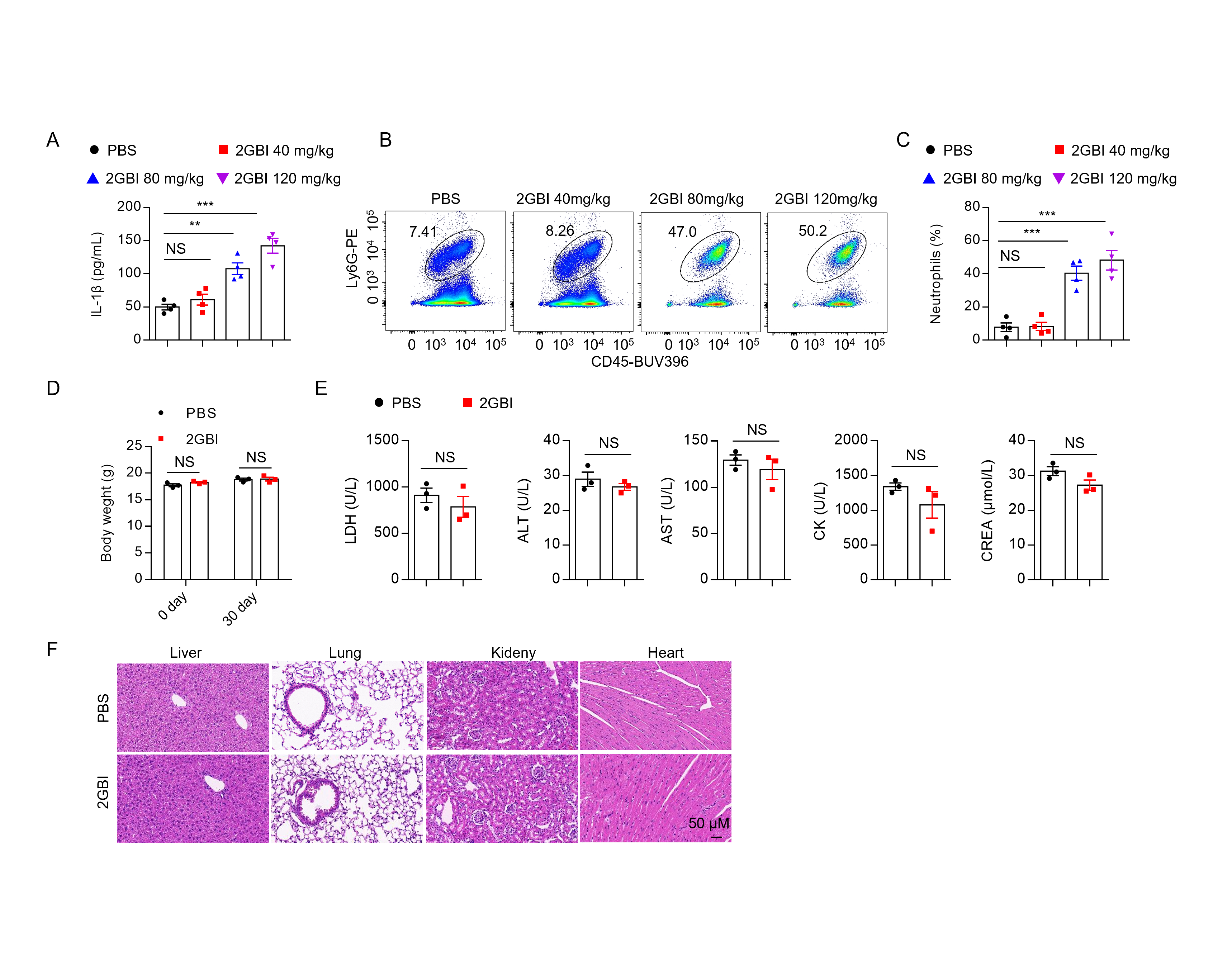


**Supplementary Fig. 10.** **2GBI has no obvious toxicity in mice, related to Fig.5.** **(A-C)** Mice were intraperitoneally injected with different concentrations of 2GBI (40 mg/kg, 80mg/kg, 120mg/kg) or PBS vehicle. Six hours later, the mice were sacrificed. **(A)** ELISA analysis of IL-1β from peritoneal lavage fluid (n = 4). Flow cytometry analysis **(B)** and quantification **(C)** of neutrophils from peritoneal lavage fluid (n = 4). **(D-F)** Mice were intraperitoneally injected with 2GBI (20 mg/kg) or PBS every two days for 30 days. **(D)** Body weight of mice (n = 3). **(E)** Quantitative analysis of lactate dehydrogenase (LDH), alanine aminotransferase (ALT), aspartate aminotransferase (AST), creatine kinase (CK), and creatinine (CREA) in the serum of mice (n = 3). **(F)** H&E histology of liver, lung, kidney, and heart. Data represent two independent experiments **(A, C, D, E)** or are typical photographs of one representative experiment **(F)**. All data are displayed by mean ± SEM. Statistical significance was analyzed by unpaired Student 's t-test: ***P* < 0.01, ****P* < 0.001, NS, no significance.
