# Supplementary material for: A small molecule directly targets NLRP3 to promote inflammasome activation and antitumor immunity
Source: Cell Death Dis. 2025 Apr 4;16(1):252. doi: 10.1038/s41419-025-07578-0 (PMC11971322; doi:10.1038/s41419-025-07578-0)
Supplement: Supplementary file 2 — Original data [file 41419_2025_7578_MOESM2_ESM.pdf]

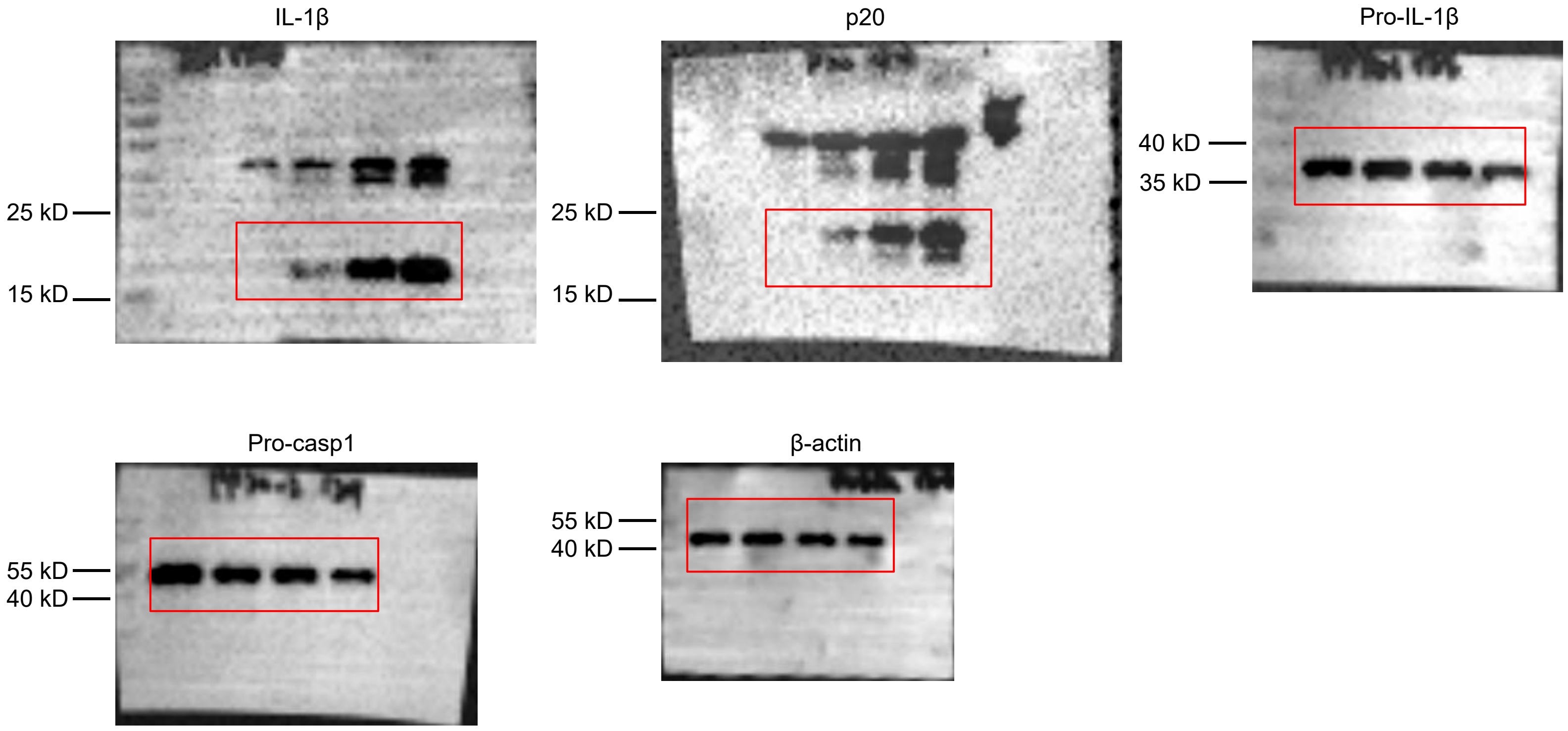
IL-1β
p20
Pro-IL-1β
25 kD
15 kD
25 kD
15 kD
40 kD
35 kD
Pro-casp1
β-actin
55 kD
40 kD
55 kD
40 kD

Fig. 1B

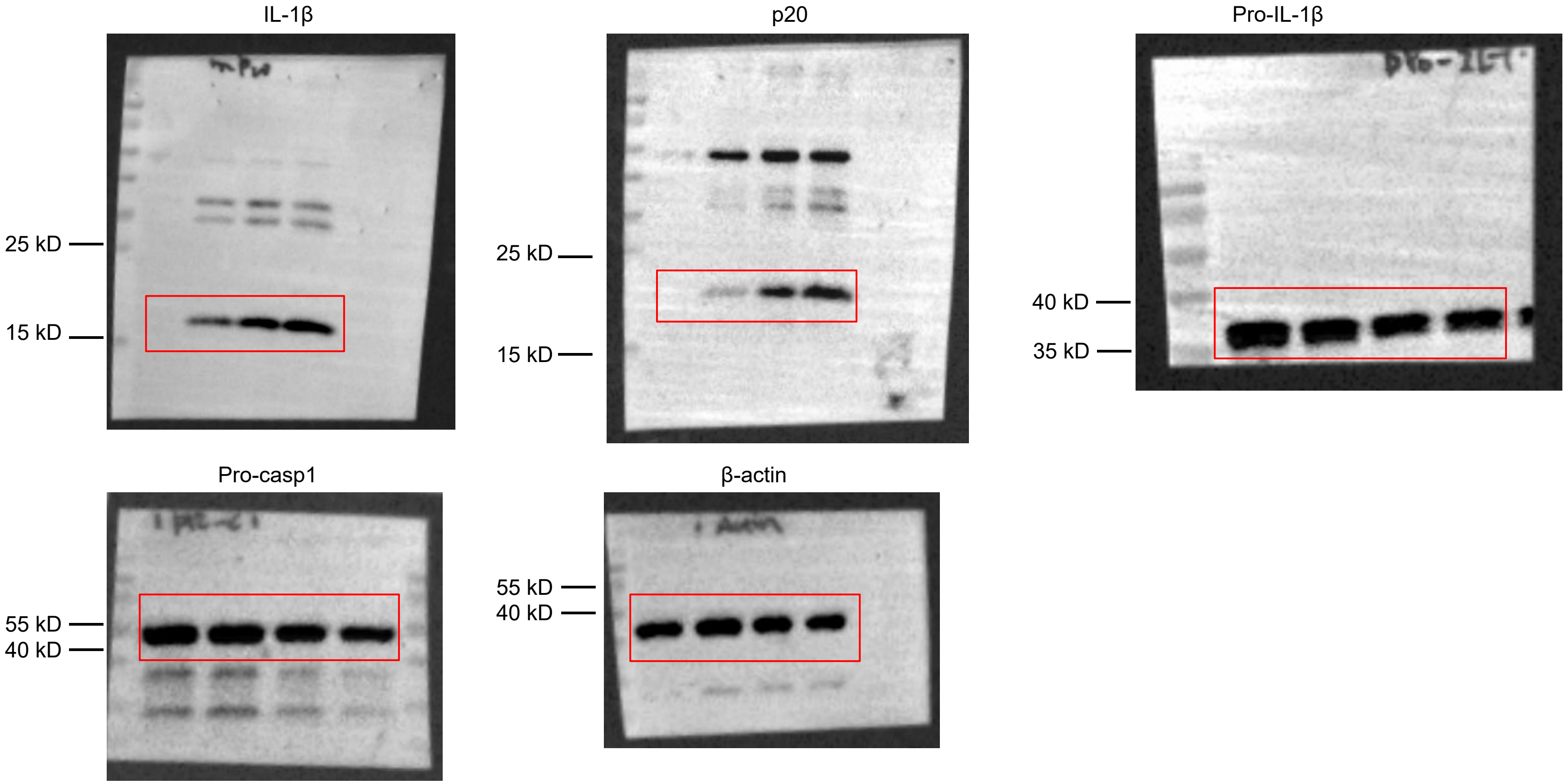
IL-1β
25 kD
15 kD
p20
25 kD
15 kD
Pro-IL-1β
40 kD
35 kD
Pro-casp1
55 kD
40 kD
β-actin
55 kD
40 kD

Fig. 1E

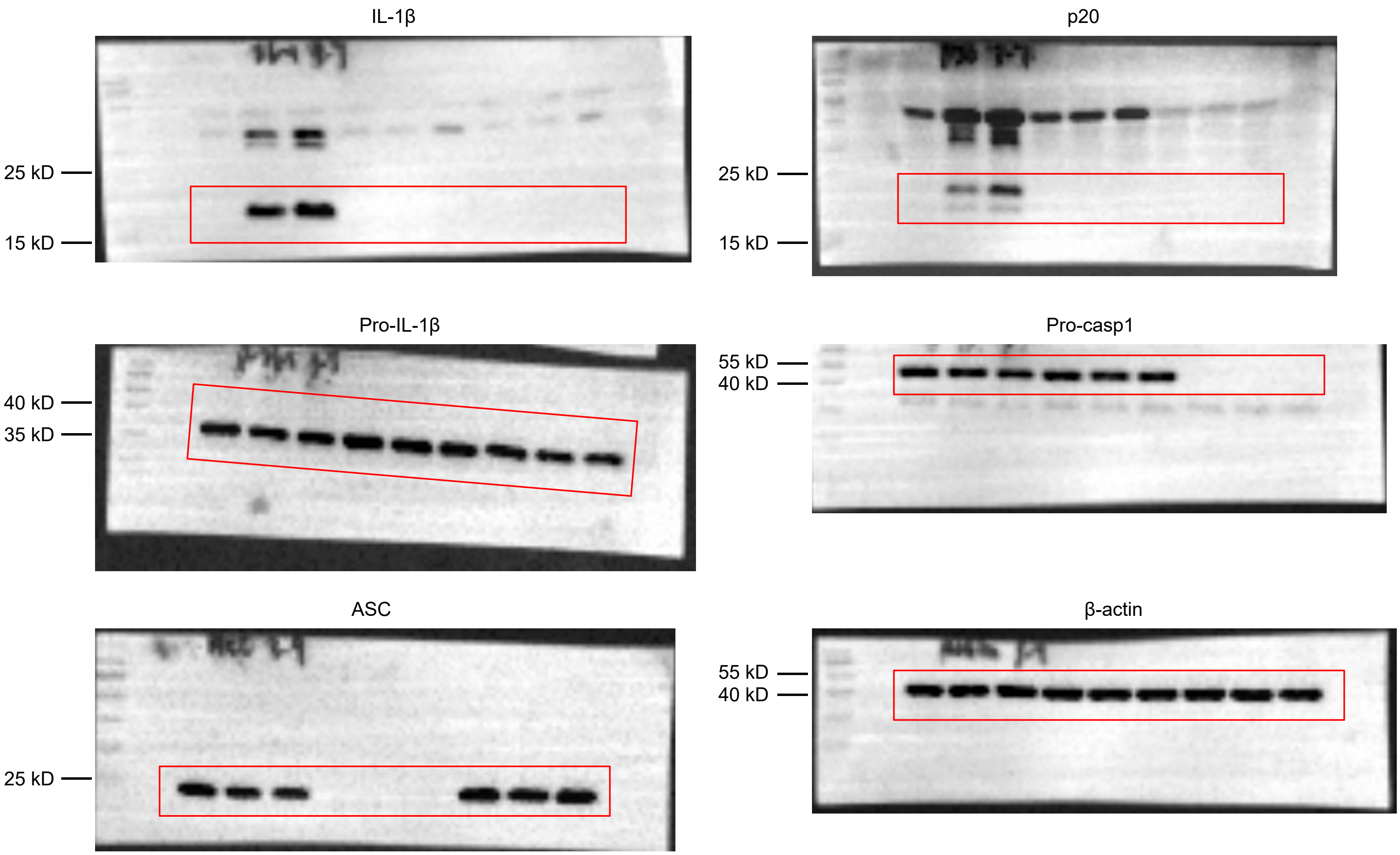
IL-1β
25 kD
15 kD
p20
25 kD
15 kD
Pro-IL-1β
40 kD
35 kD
Pro-casp1
55 kD
40 kD
ASC
25 kD
β-actin
55 kD
40 kD

Fig. 2B

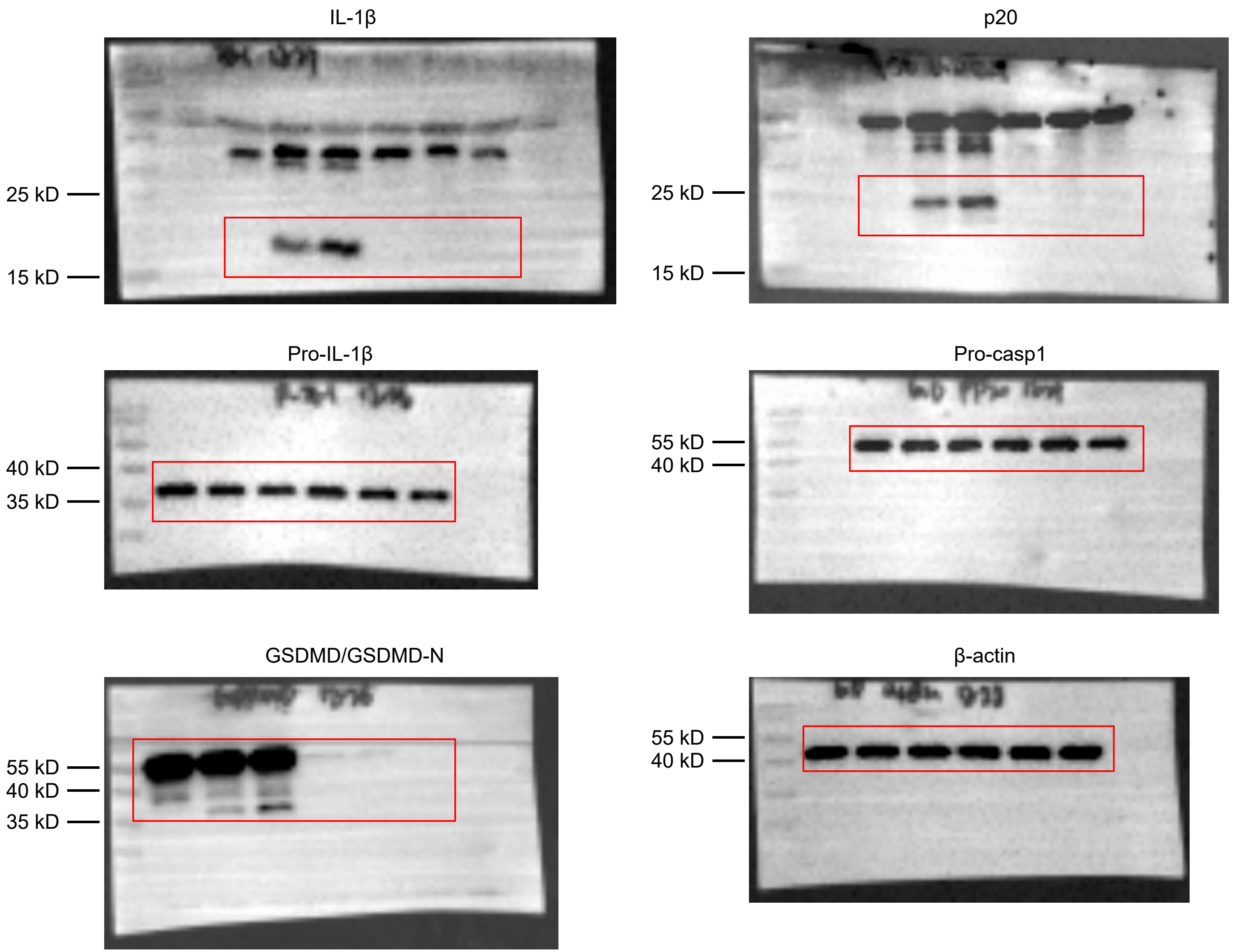
IL-1β
25 kD
15 kD
p20
25 kD
15 kD
Pro-IL-1β
40 kD
35 kD
Pro-casp1
55 kD
40 kD
GSDMD/GSDMD-N
55 kD
40 kD
35 kD
β-actin
55 kD
40 kD

Fig. 2E

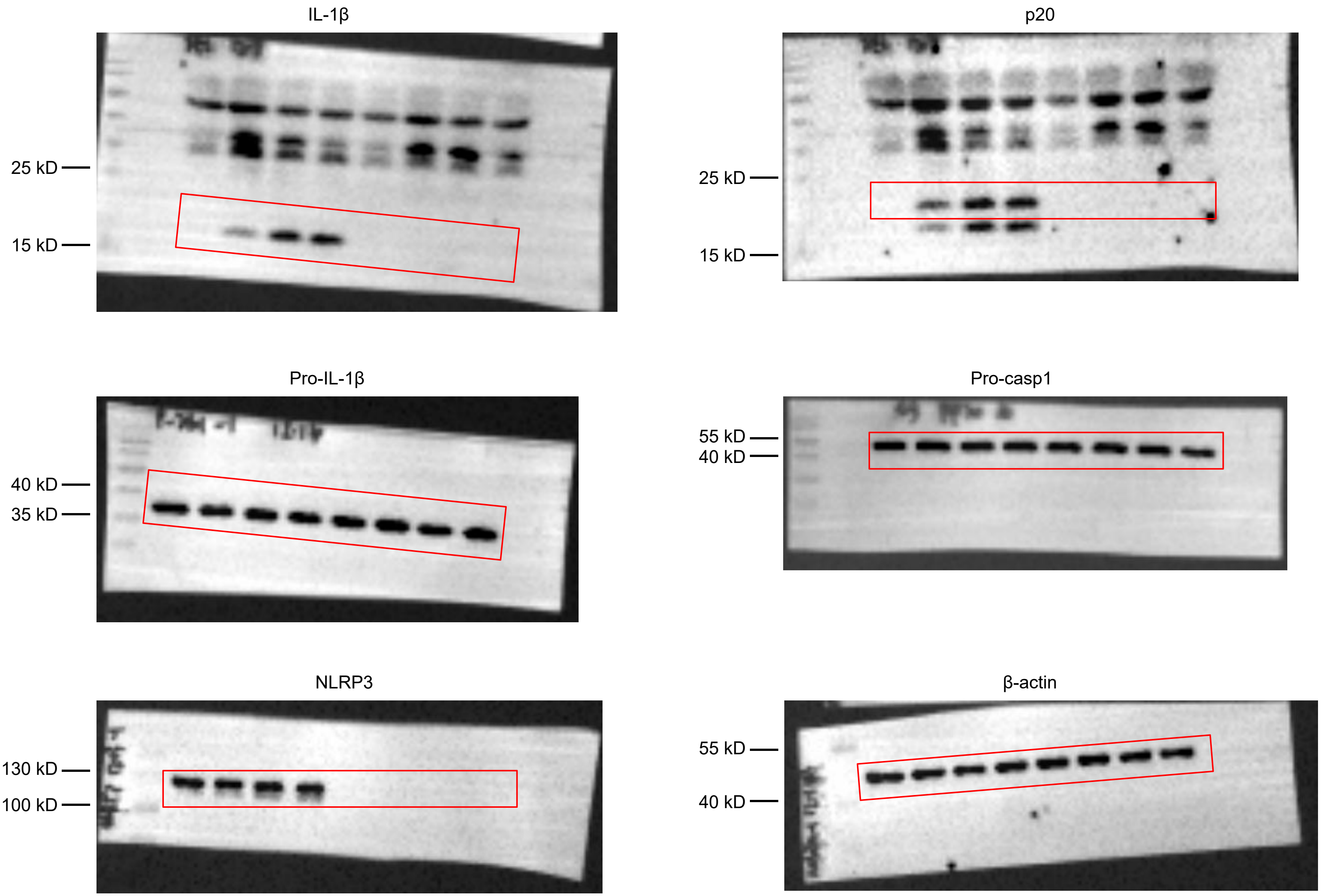
IL-1β
25 kD
15 kD
p20
25 kD
15 kD
Pro-IL-1β
40 kD
35 kD
Pro-casp1
55 kD
40 kD
NLRP3
130 kD
100 kD
β-actin
55 kD
40 kD

Fig. 2G

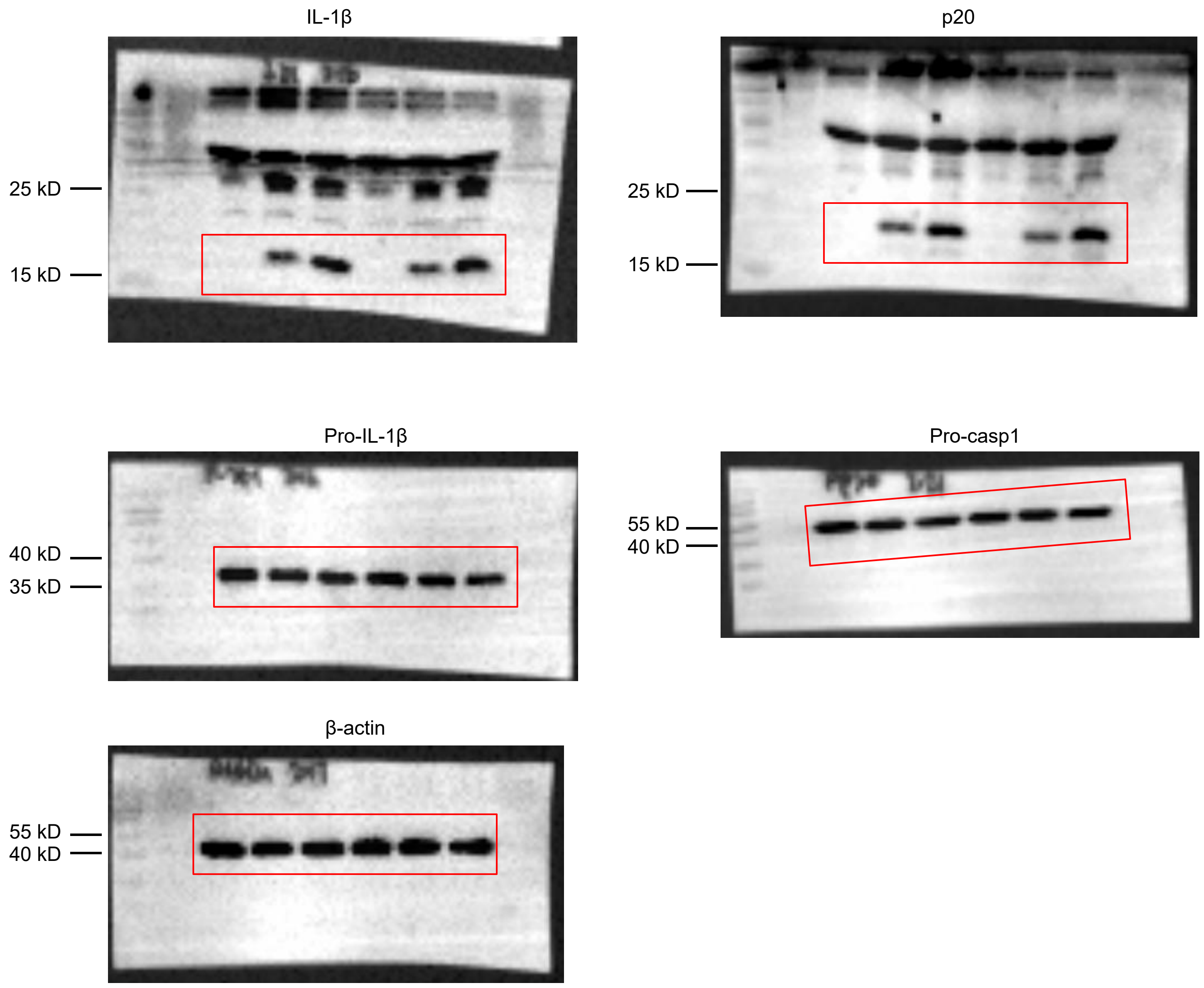
IL-1β
25 kD
15 kD
p20
25 kD
15 kD
Pro-IL-1β
40 kD
35 kD
Pro-casp1
55 kD
40 kD
β-actin
55 kD
40 kD

Fig. 3C

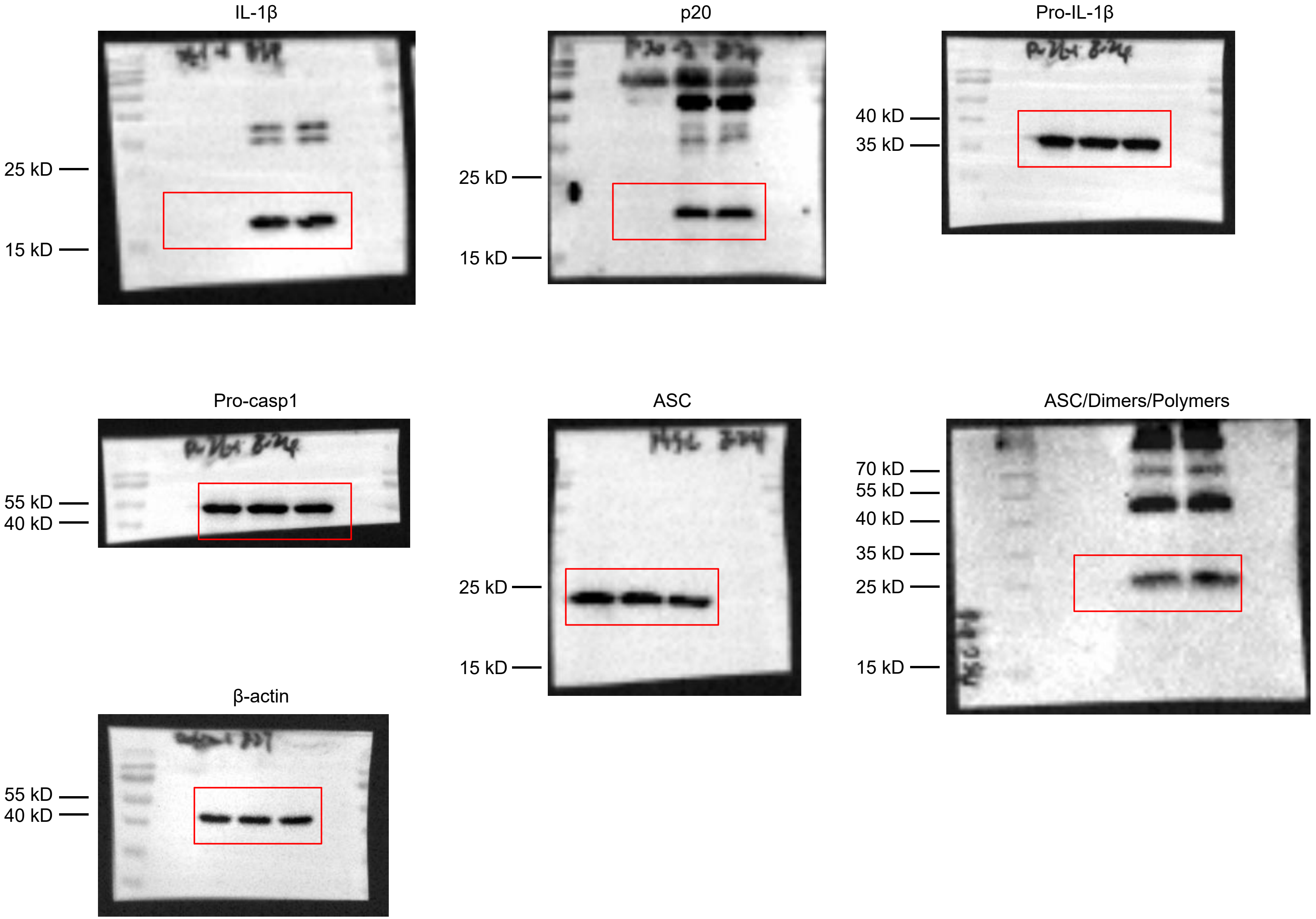
IL-1β
25 kD
15 kD
p20
25 kD
15 kD
Pro-IL-1β
40 kD
35 kD
Pro-casp1
55 kD
40 kD
ASC
25 kD
15 kD
ASC/Dimers/Polymers
70 kD
55 kD
40 kD
35 kD
25 kD
15 kD
β-actin
55 kD
40 kD

Fig. 4A

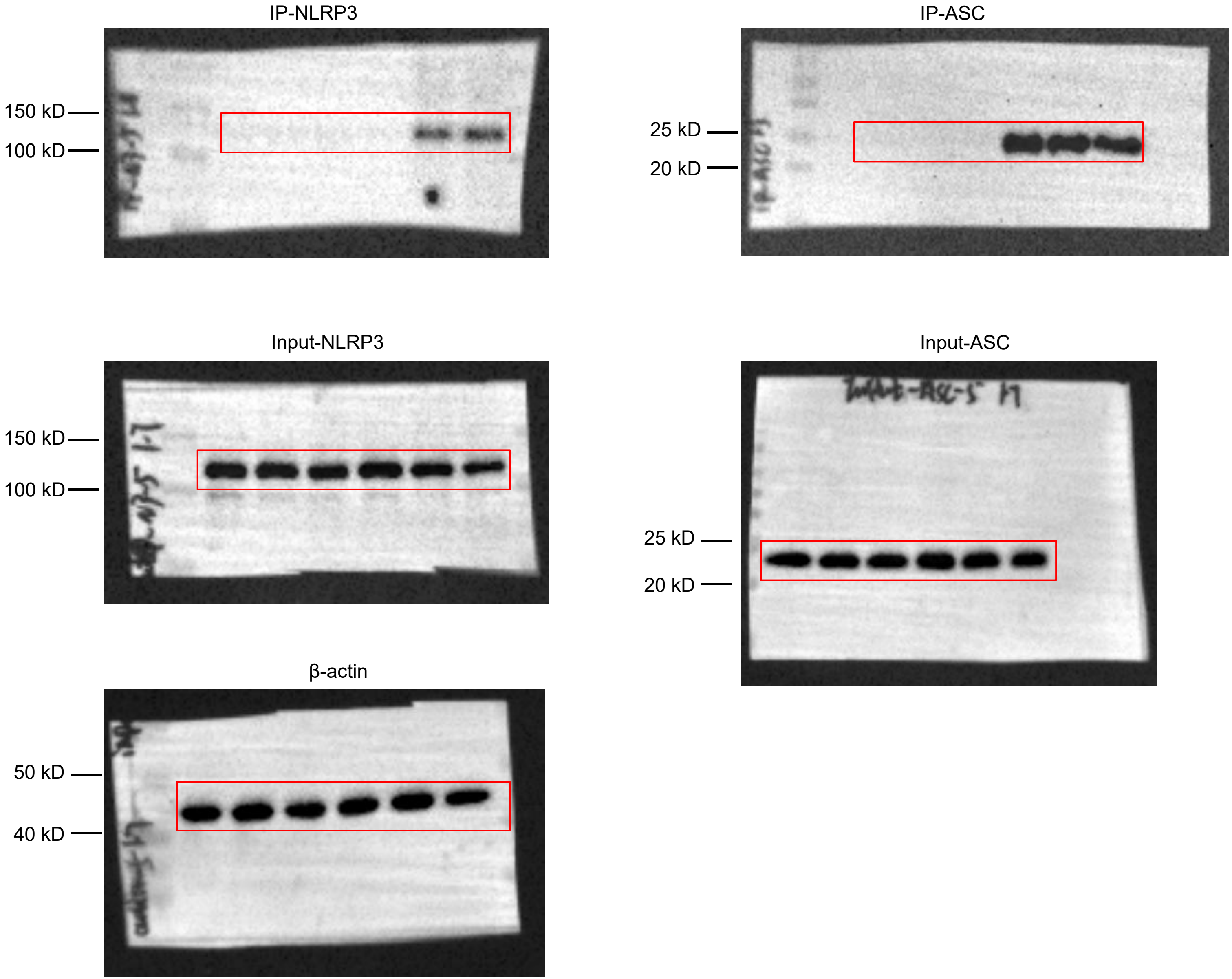
IP-NLRP3
150 kD
100 kD
IP-ASC
25 kD
20 kD
Input-NLRP3
150 kD
100 kD
Input-ASC
25 kD
20 kD
β-actin
50 kD
40 kD

Fig. 4B

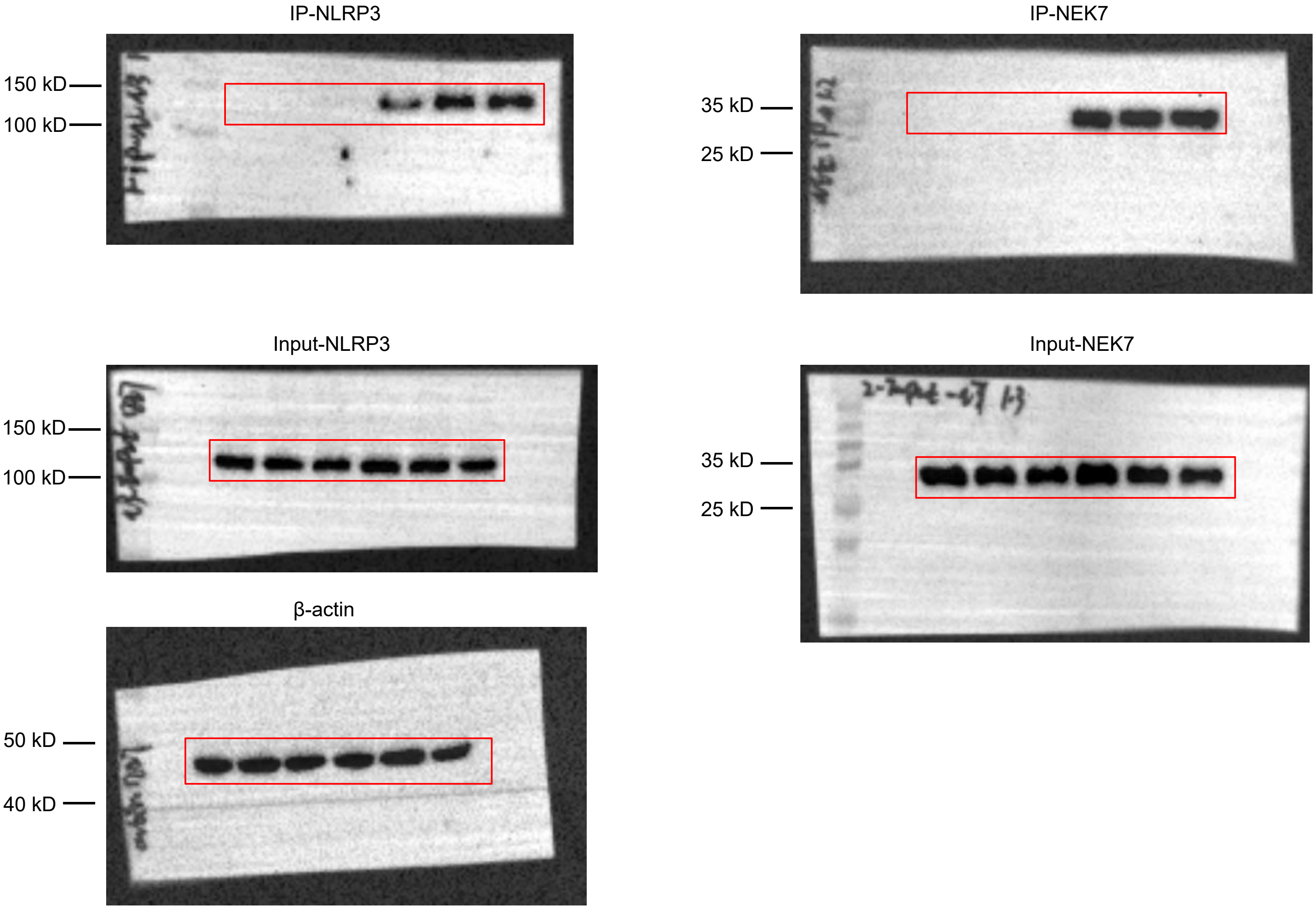
IP-NLRP3
150 kD
100 kD
IP-NEK7
35 kD
25 kD
Input-NLRP3
150 kD
100 kD
Input-NEK7
35 kD
25 kD
β-actin
50 kD
40 kD

Fig. 4C

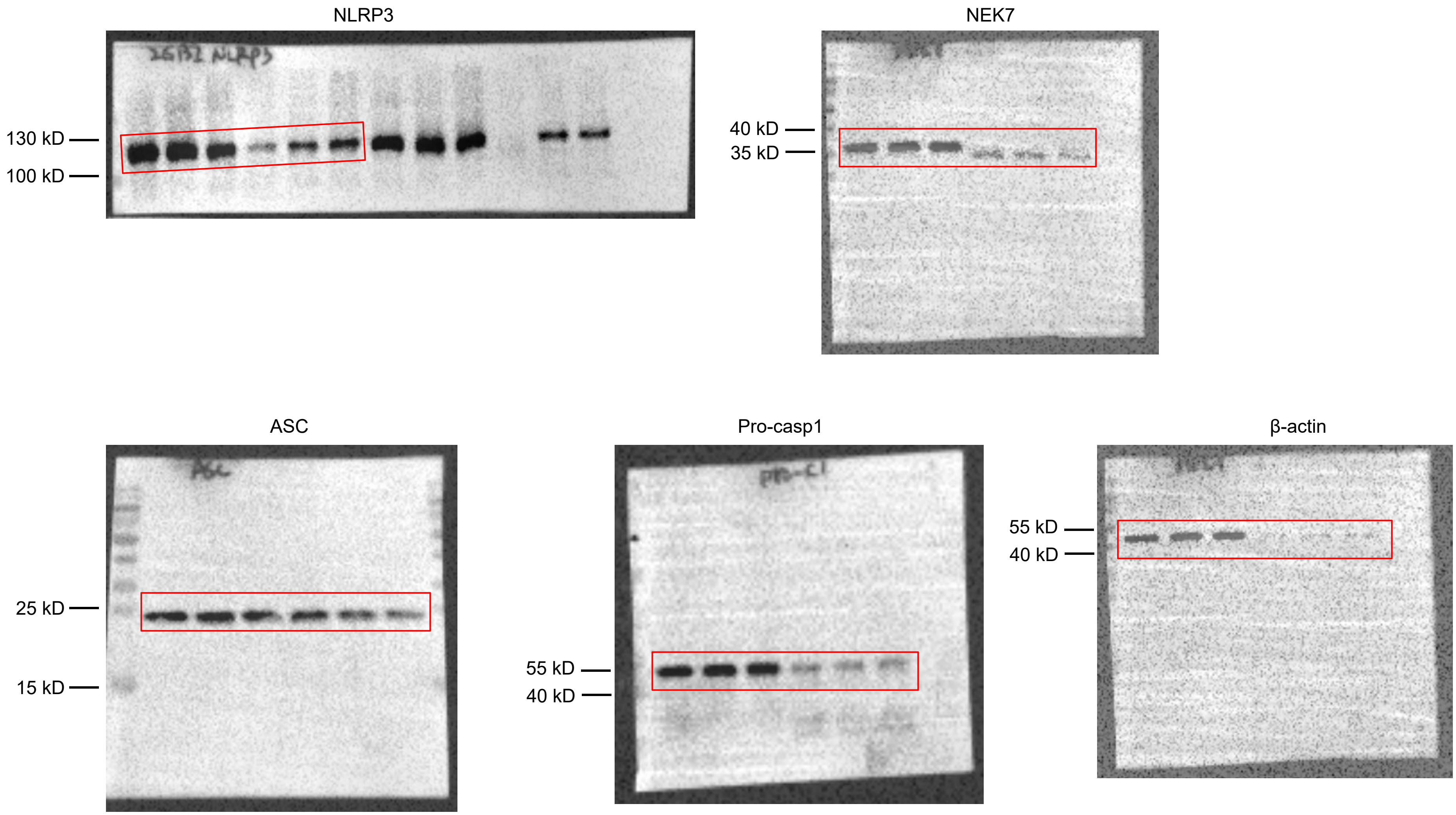
NLRP3
130 kD
100 kD
NEK7
40 kD
35 kD
ASC
25 kD
15 kD
Pro-casp1
55 kD
40 kD
β-actin
55 kD
40 kD

Fig. 4D

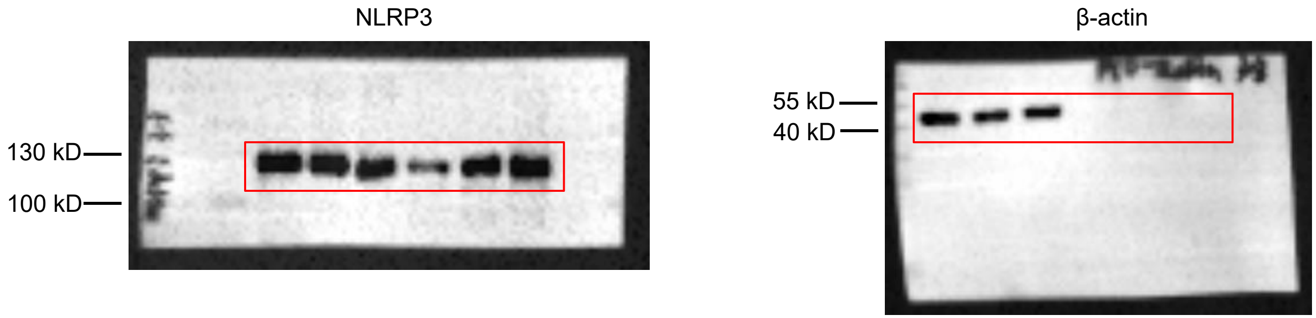

Fig. 4F

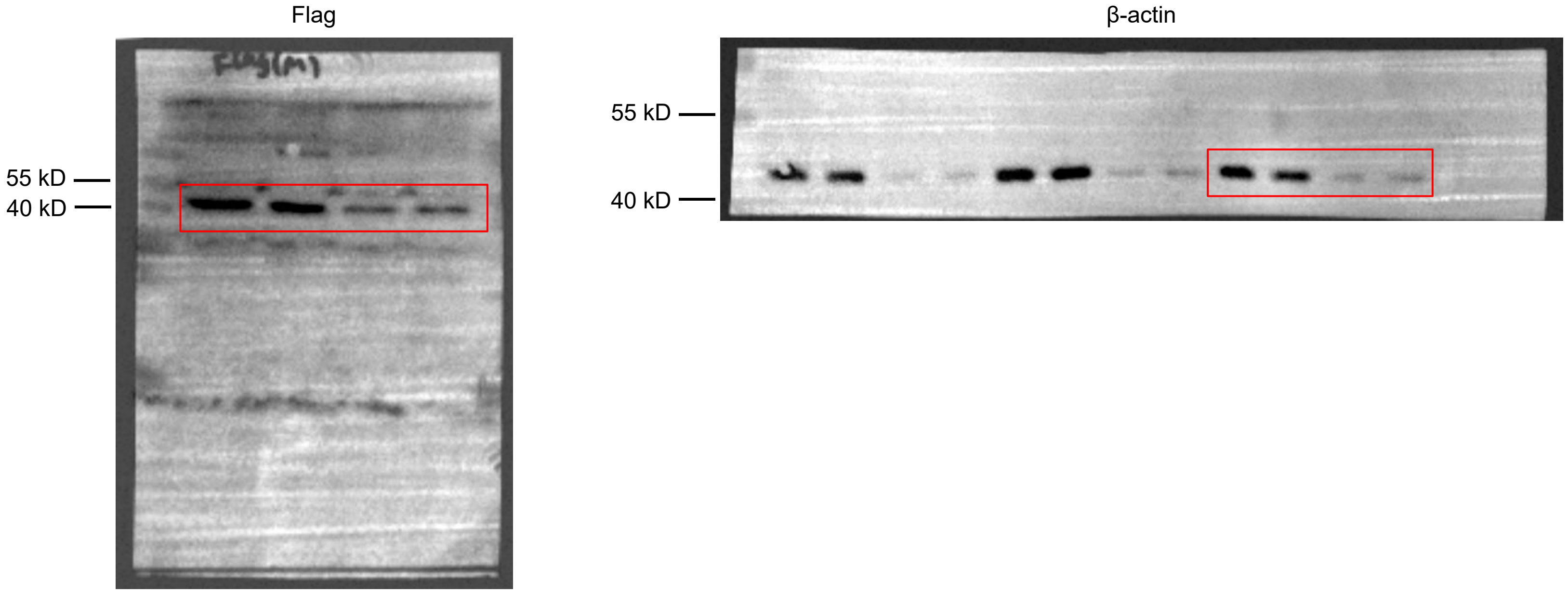

Fig. 4G

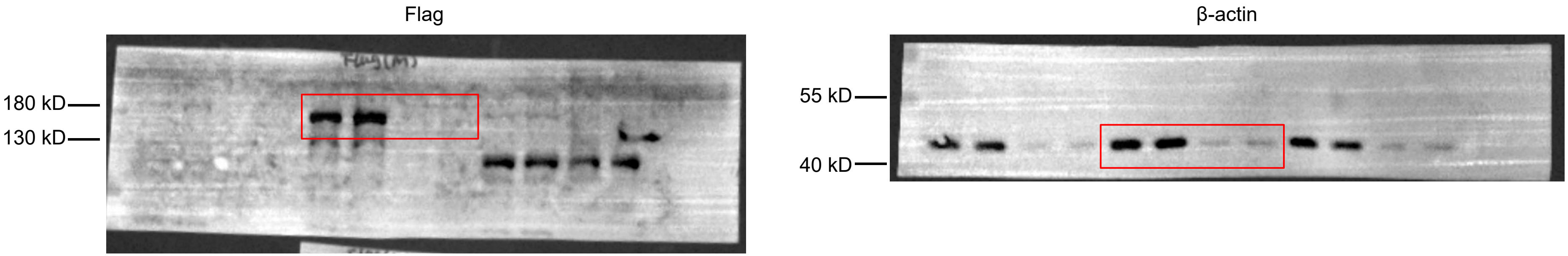

Fig. 4H

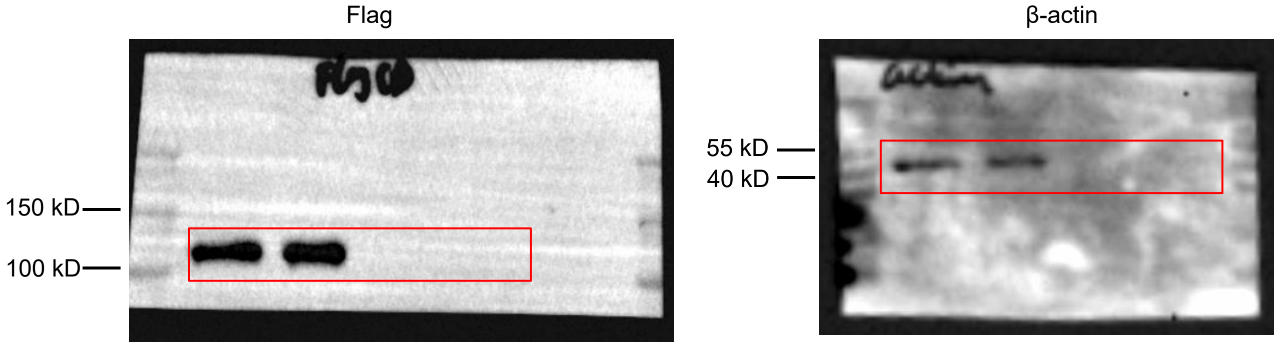

Fig. 4I

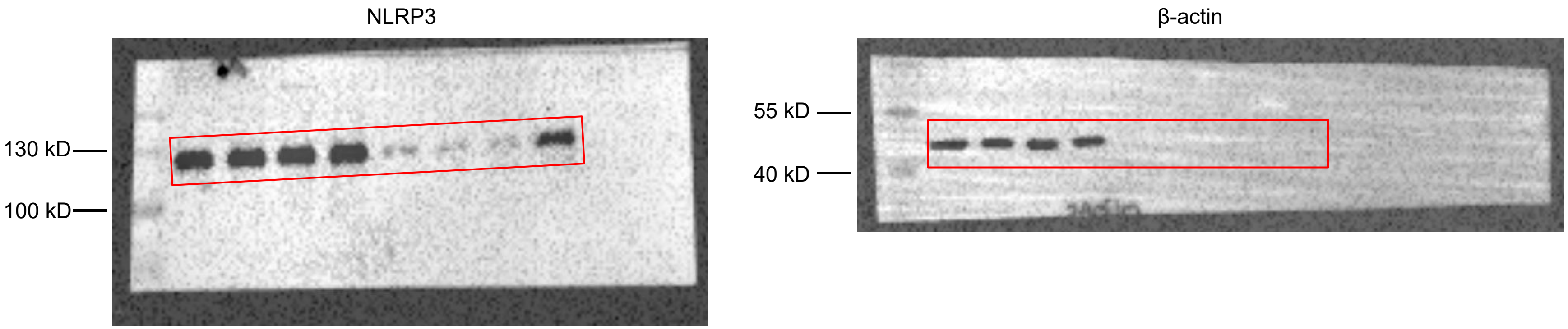

Fig. 4J

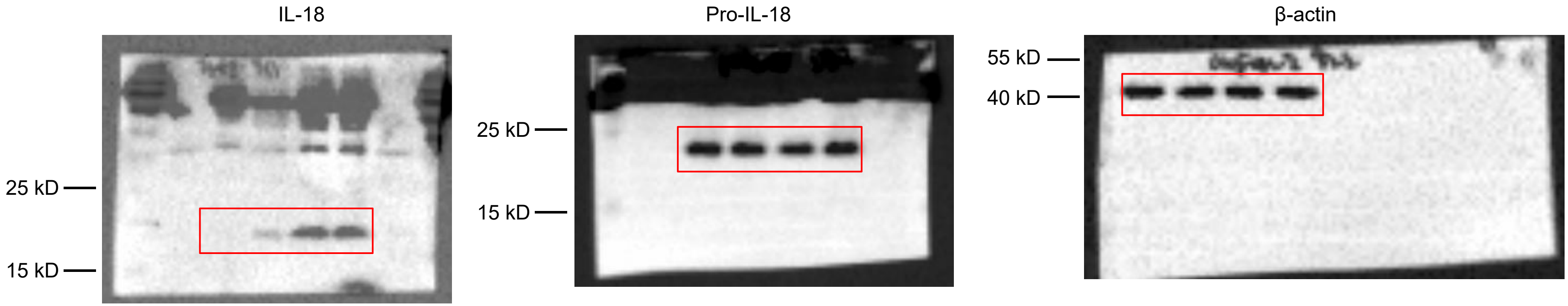

Supplementary Fig. 1B

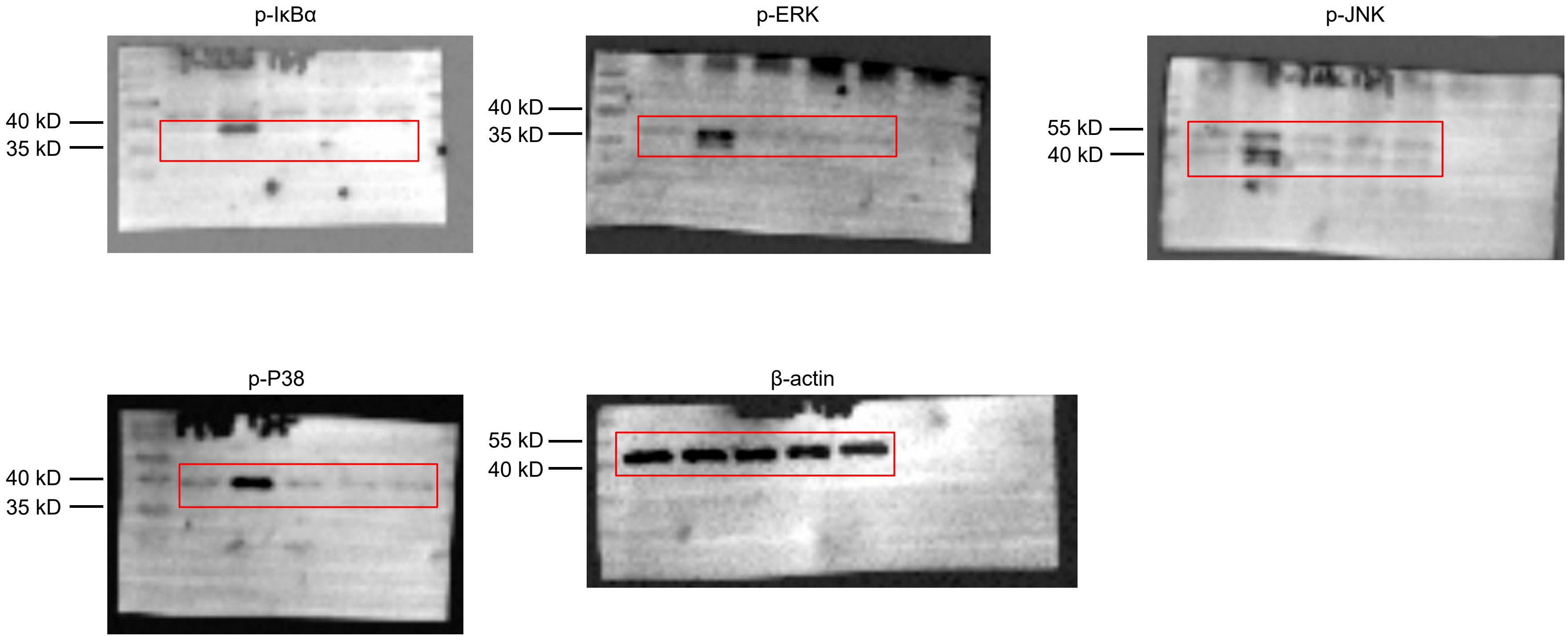

Supplementary Fig. 2C

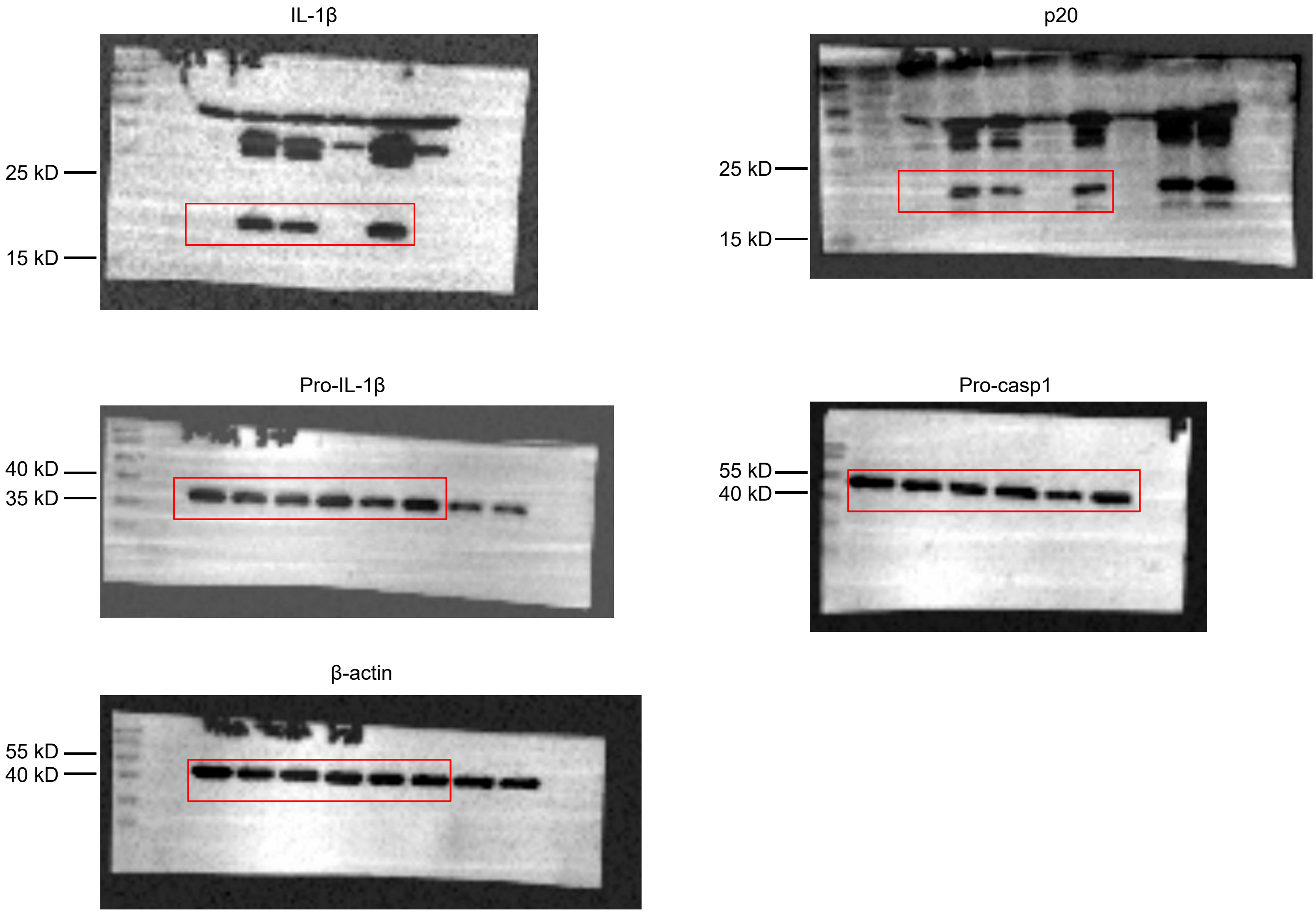

Supplementary Fig. 3C

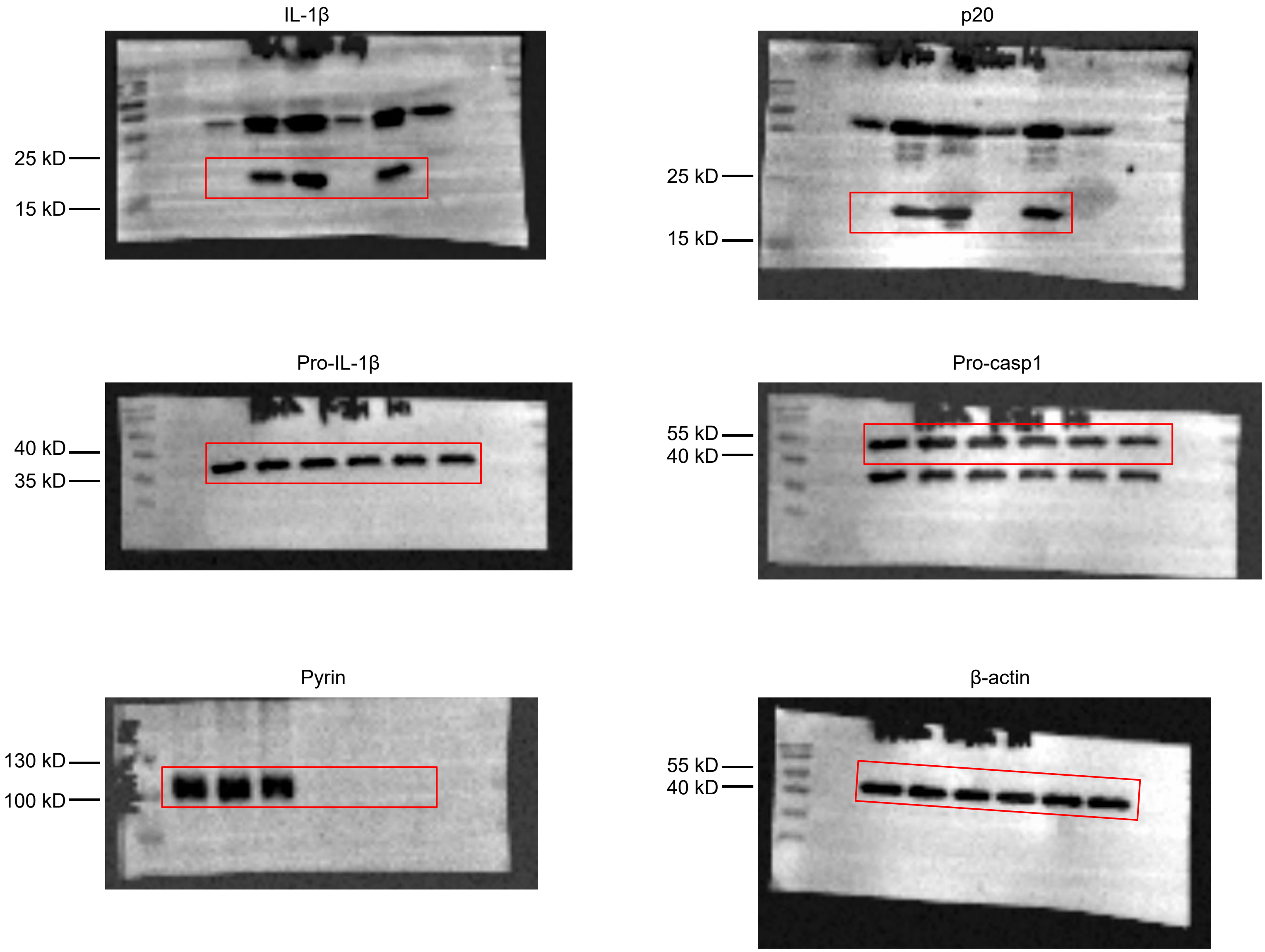

Supplementary Fig. 3E

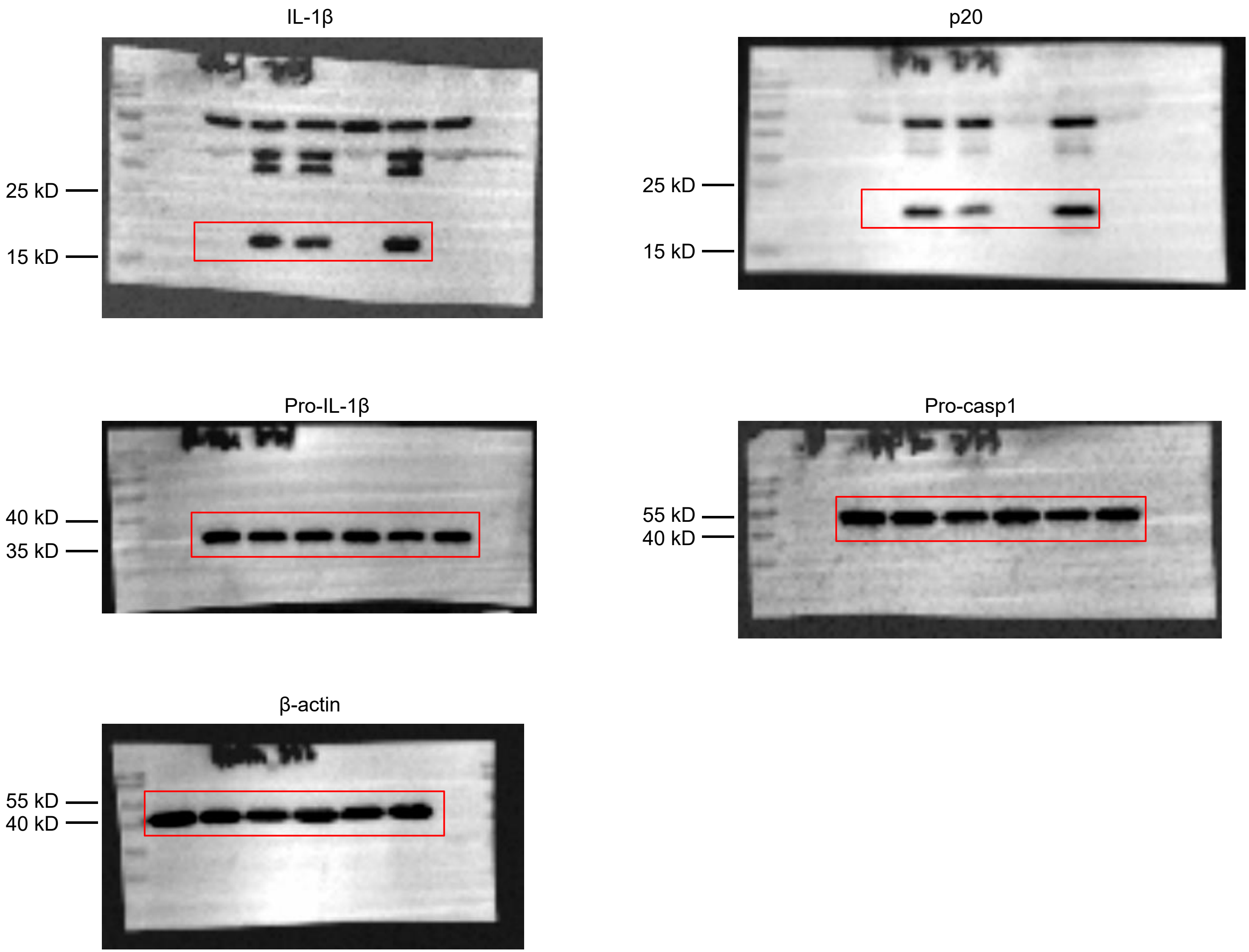

Supplementary Fig. 3G

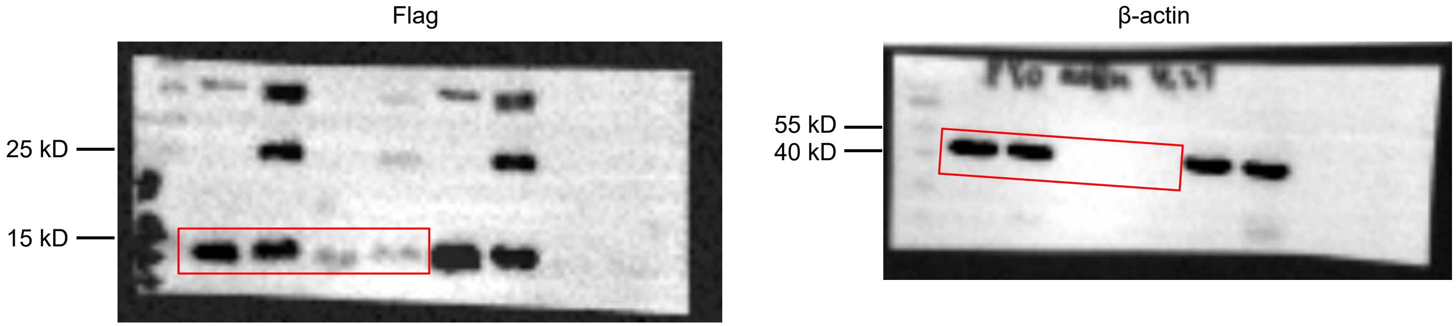

Supplementary Fig. 5A

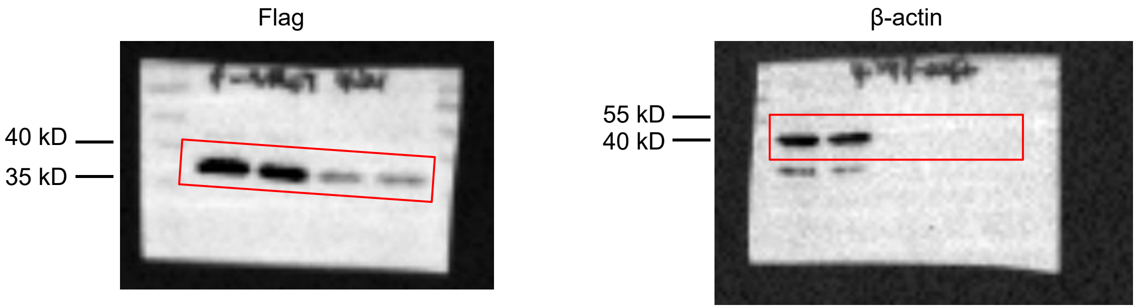

Supplementary Fig. 5B

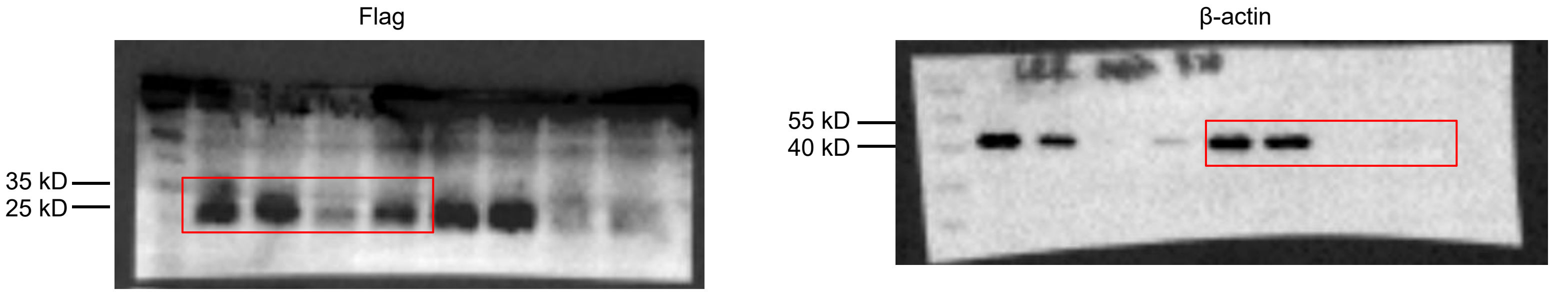

Supplementary Fig. 5C

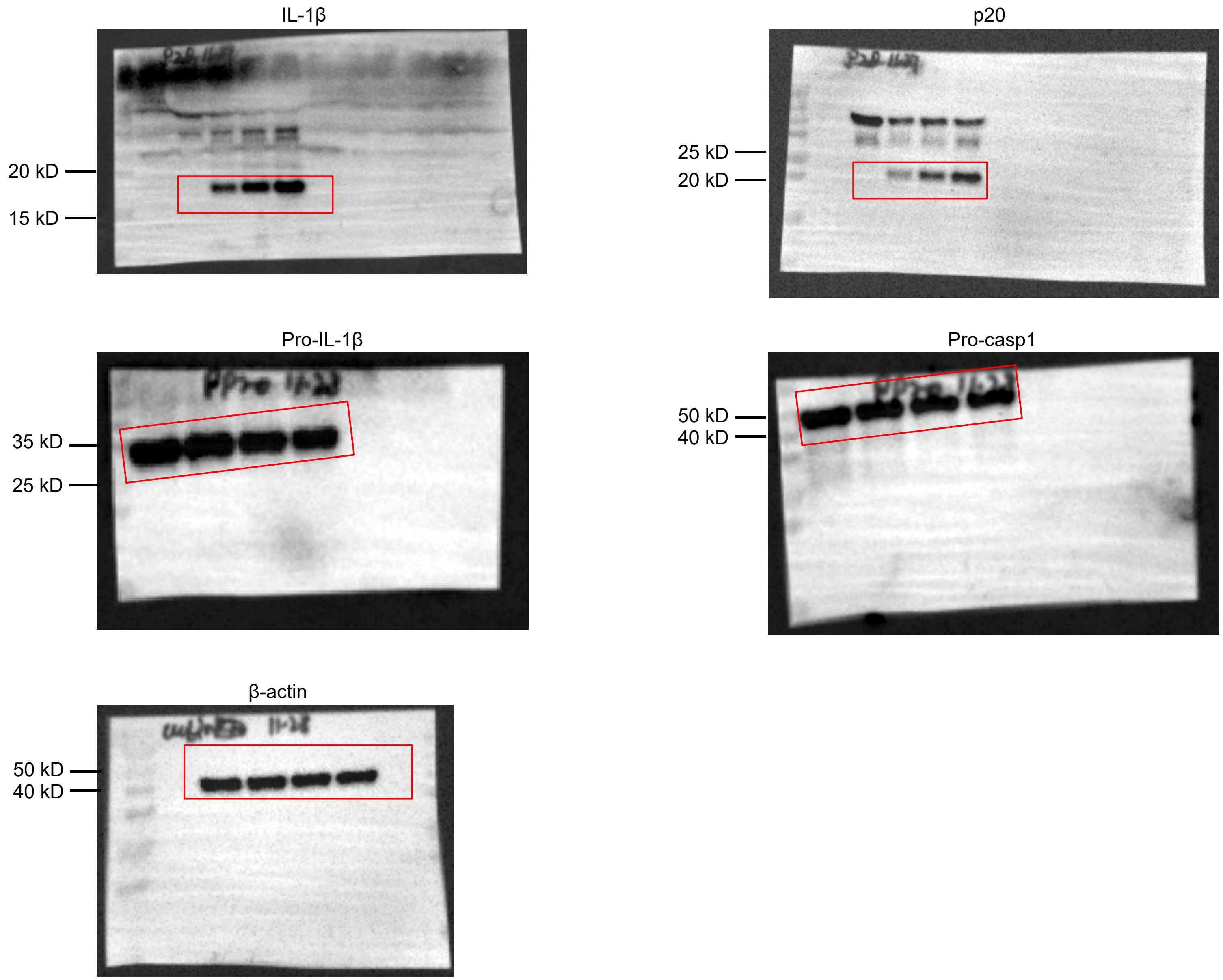

Supplementary Fig. 6B

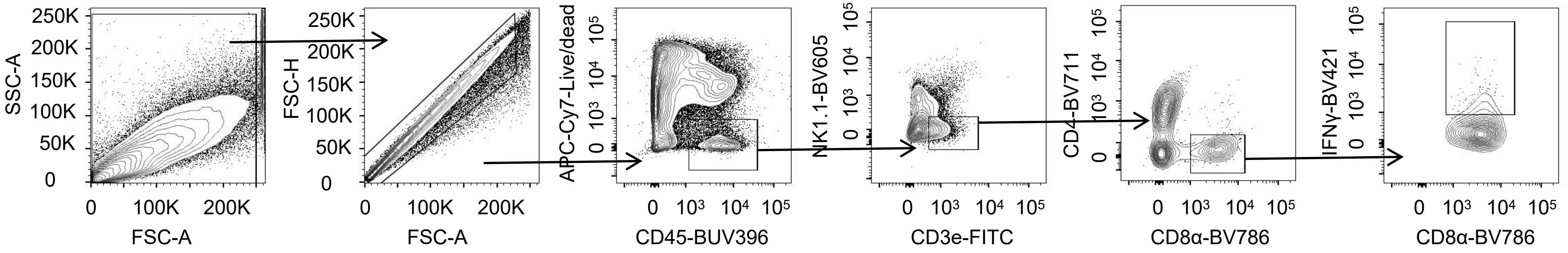

Gating strategy

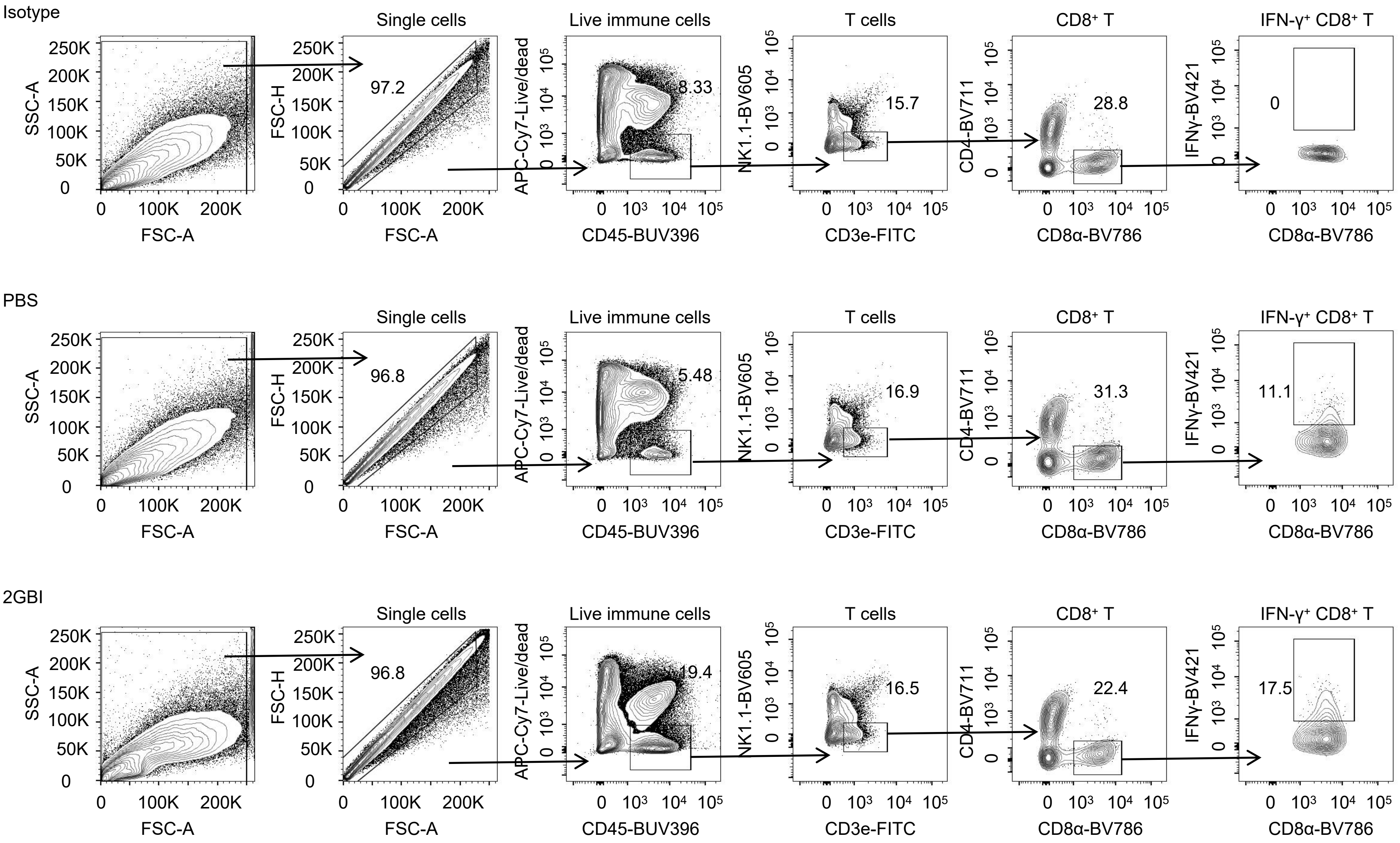

Isotype
PBS
2GBI
Single cells
Live immune cells
T cells
CD8+ T
IFN-γ+ CD8+ T
SSC-A
FSC-A
FSC-H
APC-Cy7-Live/dead
CD45-BUV396
NK1.1-BV605
CD3e-FITC
CD4-BV711
CD8α-BV786
IFNγ-BV421
97.2
8.33
15.7
28.8
0
96.8
5.48
16.9
31.3
11.1
96.8
19.4
16.5
22.4
17.5

Fig. 5L

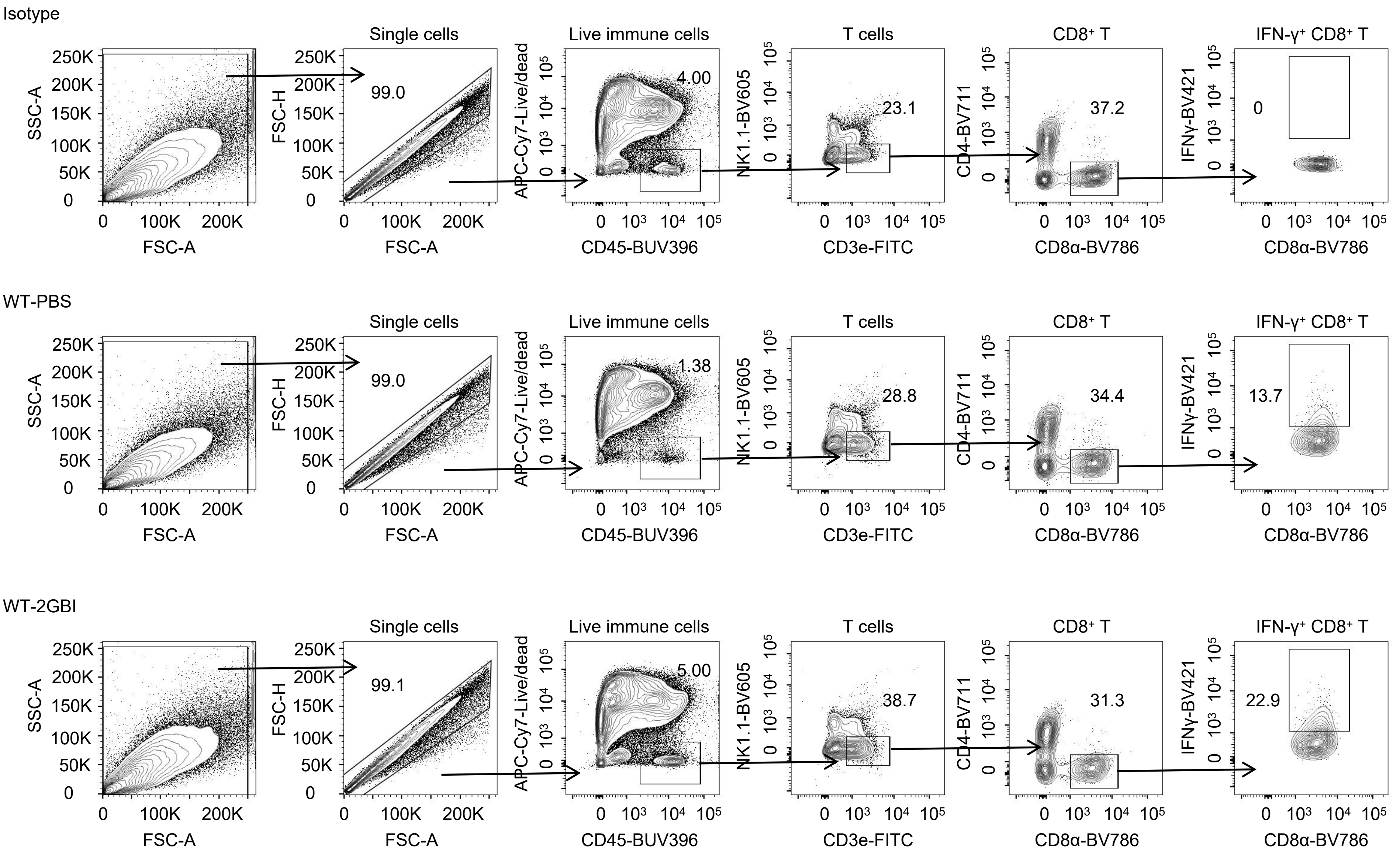

Isotype
WT-PBS
WT-2GBI
Single cells
Live immune cells
T cells
CD8⁺ T
IFN-γ⁺ CD8⁺ T
SSC-A
FSC-A
FSC-H
APC-Cy7-Live/dead
CD45-BUV396
NK1.1-BV605
CD3e-FITC
CD4-BV711
CD8α-BV786
IFNγ-BV421
99.0
4.00
23.1
37.2
0
99.0
1.38
28.8
34.4
13.7
99.1
5.00
38.7
31.3
22.9

Fig. 6H

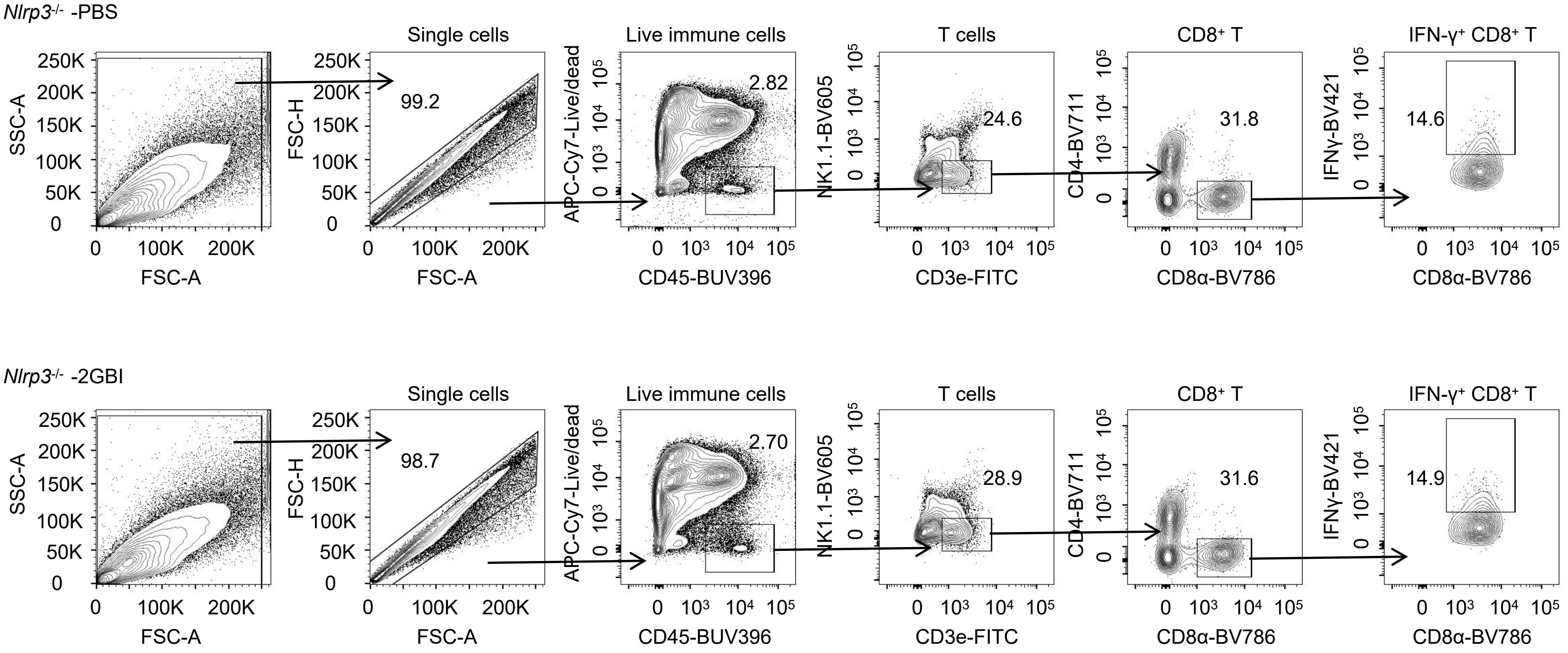
Nlrp3-/- -PBS
Single cells
Live immune cells
T cells
CD8⁺ T
IFN-γ⁺ CD8⁺ T
99.2
2.82
24.6
31.8
14.6
SSC-A
FSC-A
FSC-H
APC-Cy7-Live/dead
CD45-BUV396
NK1.1-BV605
CD3e-FITC
CD4-BV711
CD8α-BV786
IFNγ-BV421
Nlrp3-/- -2GBI
98.7
2.70
28.9
31.6
14.9

Fig. 6H
